# Monitoring melanoma patients on treatment reveals a distinct macrophage population driving targeted therapy resistance

**DOI:** 10.1016/j.xcrm.2024.101611

**Published:** 2024-06-27

**Authors:** Jelena Vasilevska, Phil Fang Cheng, Julia Lehmann, Egle Ramelyte, Julia Martínez Gómez, Florentia Dimitriou, Federica Sella, TuPro Consortium, Daria Ferretti, Adrian Salas-Bastos, Whitney Shannon Jordaan, Mitchell Paul Levesque, Reinhard Dummer, Lukas Sommer

**Affiliations:** 1 Institute of Anatomy, University of Zurich, 8057 Zurich, Switzerland; 2 Department of Dermatology, University of Zurich Hospital and Faculty of Medicine, University of Zurich, Zurich, Switzerland

**Keywords:** melanoma, targeted therapy, resistance, tumor-associated macrophages, single-cell transcriptome analysis, immune landscape, tumor microenvironment, Periostin, CD44 pathway

## Abstract

Resistance to targeted therapy remains a major clinical challenge in melanoma. To uncover resistance mechanisms, we perform single-cell RNA sequencing on fine-needle aspirates from resistant and responding tumors of patients undergoing BRAFi/MEKi treatment. Among the genes most prominently expressed in resistant tumors is POSTN, predicted to signal to a macrophage population associated with targeted therapy resistance (TTR). Accordingly, tumors from patients with fast disease progression after therapy exhibit high POSTN expression levels and high numbers of TTR macrophages. POSTN polarizes human macrophages toward a TTR phenotype and promotes resistance to targeted therapy in a melanoma mouse model, which is associated with a phenotype change in intratumoral macrophages. Finally, polarized TTR macrophages directly protect human melanoma cells from MEKi-induced killing via CD44 receptor expression on melanoma cells. Thus, interfering with the protective activity of TTR macrophages may offer a strategy to overcome resistance to targeted therapy in melanoma.

## Highlights

• Targeted therapy resistance is associated with high expression of POSTN in melanoma
• POSTN induces a targeted therapy resistance (TTR)-associated macrophage phenotype
• POSTN promotes therapy resistance in a mouse melanoma model
• TTR macrophages orchestrate melanoma resistance formation via CD44 signaling

## Graphical abstract

**Figure:**
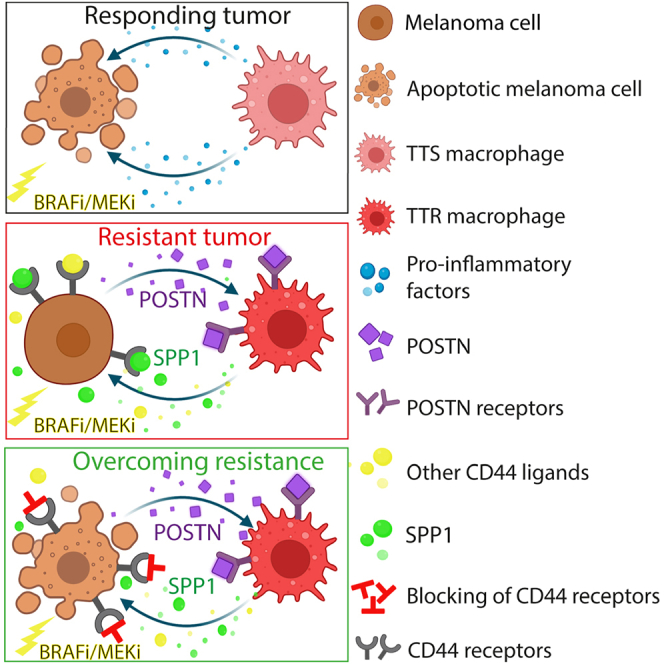

## Teaser

Vasilevska et al. show that expression of the secreted factor POSTN fosters targeted therapy resistance in melanoma by promoting the emergence of a tumor-cell-protective macrophage population.

## Introduction

Despite the groundbreaking outcomes achieved in the understanding and treatment of metastatic melanoma, it remains one of the most aggressive cancers worldwide. More than 50% of melanomas carry mutations in the mitogen-activated protein kinase (MAPK) pathway, with oncogenic alterations in BRAF and NRAS being the most prevalent.^1^^,^^2^ Therefore, substantial efforts have been made to develop selective BRAF inhibitors in combination with MEK inhibitors.^3^^,^^4^ Although these drugs are associated with pronounced clinical benefits, approximately 50% of the responders develop acquired resistance after a median of 6–12 months.^5^ Therefore, early molecular characterization of high-risk stage II, III, and IV tumors in melanoma patients has become a research priority to define the factors driving resistance.^6^

Applying next-generation sequencing techniques, many genetic and non-genetic mechanisms of adaptive resistance to BRAF and MEK inhibitors have been identified.^7^^,^^8^^,^^9^ Tsoi et al.^10^ demonstrated that the regulation of melanoma resistance proceeds via stepwise de-differentiation of four distinct cell subtypes: undifferentiated, neural crest-like, transitory, and melanocytic cells. These data revealed the high plasticity of melanoma cells and indicated that different phenotypic states could contribute to intratumoral melanoma heterogeneity and therapeutic response. The existence of specific, intrinsically therapy-resistant melanoma subpopulations suggests new potential vulnerabilities that could be targeted, for instance in the context of combinatorial treatment strategies. Indeed, recent studies have demonstrated that in cell culture or in patient-derived xenograft (PDX) models, targeting de-differentiated melanoma cells could overcome resistance induced by BRAFi/MEKi or immunotherapy-associated cytokines.^9^^,^^11^^,^^12^

To understand how molecular features of drug resistance are acquired during targeted therapy, we acquired longitudinal tumor cell collections by fine-needle aspiration (FNA) from four consenting melanoma patients at baseline and at different time points following BRAFi/MEKi treatment, followed by single-cell RNA sequencing (scRNA-seq). Gene expression analysis revealed POSTN as the factor most prominently associated with resistance to therapy. Together with the analysis of further patient cohorts and functional assays, our study revealed that high POSTN expression was associated with infiltration and polarization of a targeted therapy resistance (TTR)-associated macrophage population that mediates resistance formation.

## Results

### Screening for targeted therapy resistance mechanisms using FNA biopsies from patients on treatment

**Figure 1:**
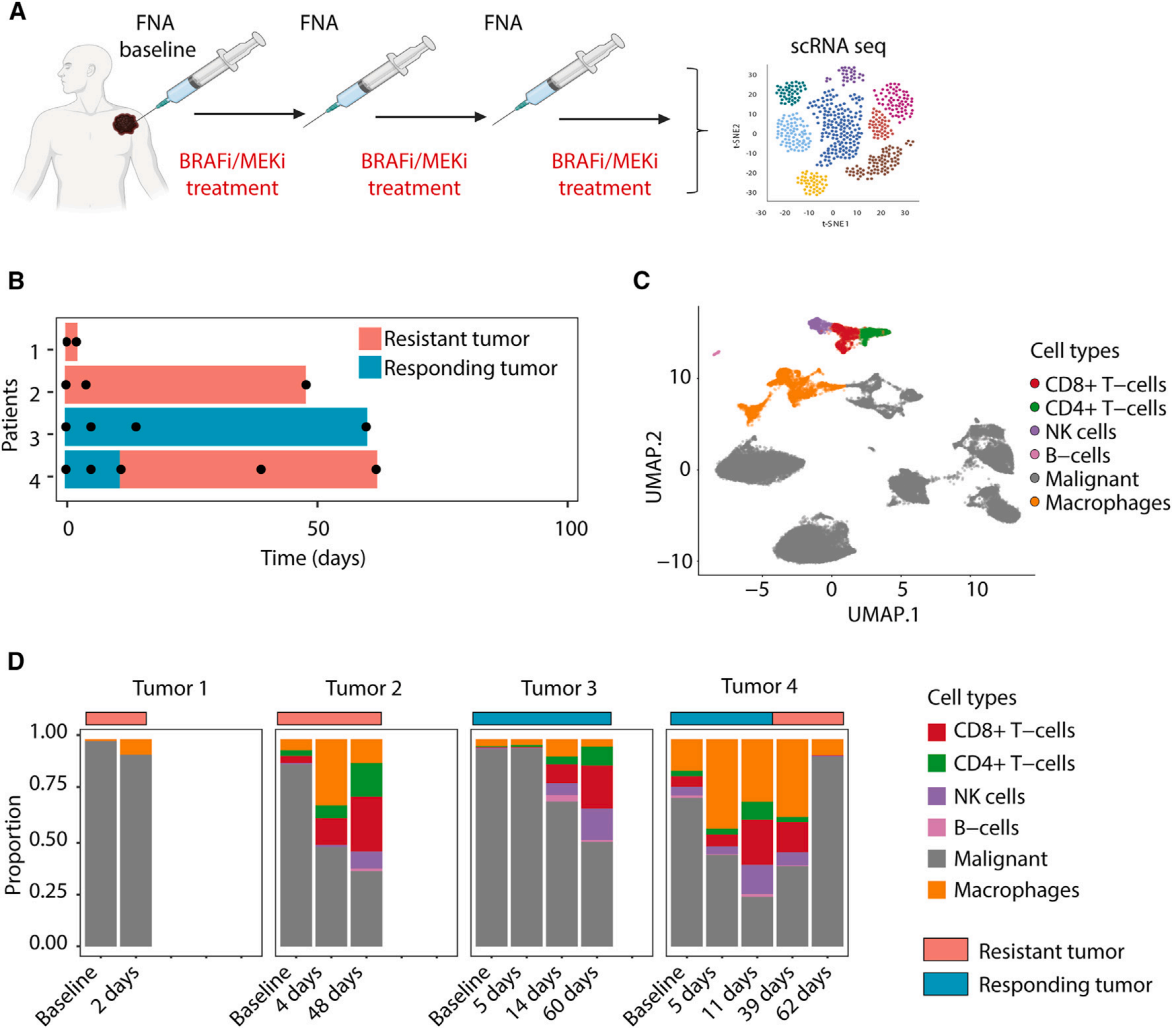
Experimental setup and single-cell transcriptomics landscape of melanoma patients (A) Schematic overview of cohort 1. Fine-needle aspiration (FNA) biopsies were obtained from patients before treatment (baseline) and at subsequent time points during treatment with targeted therapy. Cohort 1 includes 4 melanoma patients. (B) Swimmer’s plot showing the timeline and therapy response for 4 melanoma patients. Each dot represents the time at which an FNA biopsy was collected. Blue represents tumors responding to therapy and orange represents tumors resistant to the therapy. (C) Uniform Manifold Approximation and Projection (UMAP) plot for cell populations obtained from all the FNAs. (D) Cell-type diversity plots of identified cell populations for each tumor in cohort 1 upon targeted therapy course.

To perform longitudinal monitoring of melanoma patients on treatment and to investigate how the cellular landscape dynamically changes in response to targeted therapy, we applied a longitudinal FNA cellular collection method—cohort 1 (Figure 1A).^13^ Applying scRNA-seq technology, we analyzed FNAs from four consenting melanoma patients before dabrafenib and trametinib (BRAFi/MEKi) treatment and at different time points following treatment (Figure 1B). Two tumors from patients 1 and 2, respectively, turned out to be therapy resistant. Tumor 3 from patient 3 demonstrated a complete response, while tumor 4 from patient 4 responded in the beginning without reaching a complete response and progressed later. All patients had different treatment histories before receiving BRAFi/MEKi therapy (Table S1).

The scRNA-seq analysis revealed 31 cell clusters in our samples (Figure S1A). The identified cell types were CD8^+^ T cells, CD4^+^ T cells, NK cells, B cells, macrophages, and melanoma cells (Figure 1C). Malignant cells were inferred to have copy number aberrations compared to normal cell types (Figure S1B). Notably, no stromal cells (fibroblasts and endothelial cells) were detected in these samples. Longitudinal analysis of the individual biopsies revealed that the cell-type composition gradually changed during the treatment course (Figure 1D), where the greatest changes involved infiltration of immune cells.

### Melanoma cell populations of BRAFi/MEKi-resistant tumors display high *POSTN* and *MDK* expression in patients

We first focused on malignant cells from the four melanoma tumors. A total of 45,916 malignant cells were compared between the responders and resistant groups to identify transcriptomic differences. The malignant cells from the different patients clustered separately, revealing a high degree of patient-specific heterogeneity (Figure S1C). To investigate whether the transcriptomic profiles of malignant cells in resistant tumors overlap with resistance-related cellular phenotypes previously identified in cell culture and in PDX model systems, we applied AddModuleScore from Seurat using melanoma signatures from Hoek,^14^ Tirosh,^8^ Tsoi,^10^ Rambow,^9^ and Verfaillie.^15^ Although some cells displayed high positive enrichment scores with known melanocytic/proliferative signatures, there was a lack of enrichment for known drug-resistant signatures (Figure S1D).

**Figure 2:**
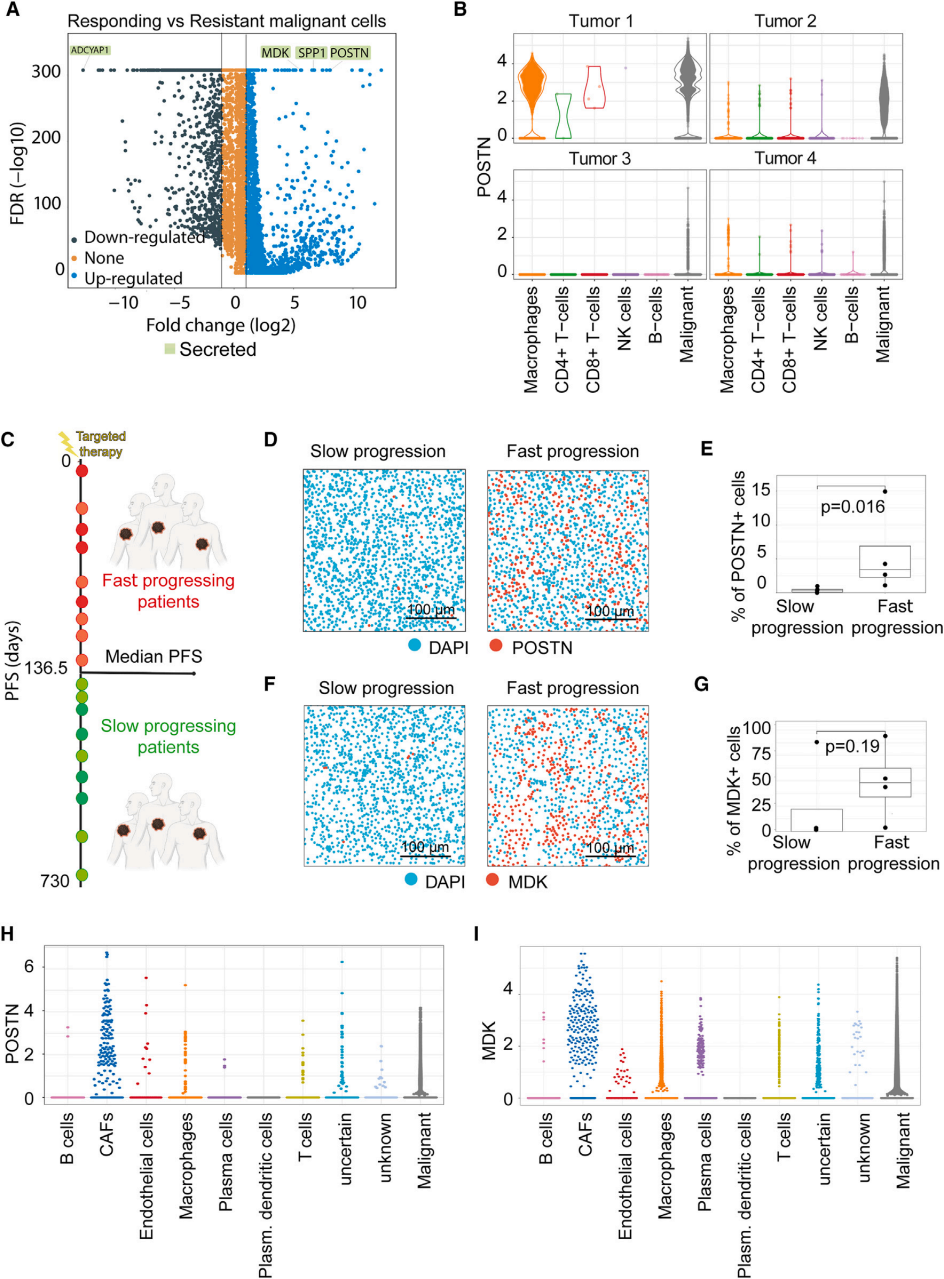
POSTN and MDK expression in resistant and responding patients (A) Volcano plot of differentially expressed genes in malignant cells between resistant tumors 1 and 2 and responding tumor 3 of cohort 1. (B) POSTN expression between tumors of patient cohort 1 and cell populations identified with scRNA-seq. (C) Schematic overview of cohort 2. Patients were treated with targeted therapy and assigned according to their progression-free survival (PFS) either to the fast (PFS ≤136.5 days) or slow (PFS ≥136.5 days) progressing subcohort (*n* = 18 patients). (D) Representative digital pictures of POSTN RNAscope staining of a fast- and slow-progressing tumor from melanoma patients in cohort 2. Blue represents cell nuclei, and red represents POSTN^+^ cells. Scale bar 100 μm (E) Proportional quantification of POSTN^+^ cells revealed by RNAscope staining (*n* = 4–5 melanoma tumors, *p* = 0.016, Wilcoxon rank-sum test). (F) Representative digital pictures of MDK RNAscope staining from melanoma patients in cohort 2. Blue represents cell nuclei, and red represents MDK^+^ cells. Scale bar 100 μm (G) Proportional quantification of MDK^+^ cells revealed by RNAscope staining (*n* = 4 melanoma tumors, *p* = 0.19, Wilcoxon rank-sum test). (H and I) POSTN (H) and MDK (I) expression in different cell populations detected in targeted therapy-resistant biopsies (*n* = 10 resistant melanoma tumors, Tumor Profiler data).

To identify genes potentially associated with drug resistance, we performed differential expression analyses between the responding (tumor 3) and resistant (tumors 1 and 2) malignant cells. Of the top 10 genes overexpressed in the resistant cells, three were secreted factors: *SPP1*, *MDK*, and *POSTN* (Figures 2A and S2A), which were also expressed in other cells of the FNA samples (Figures 2B and S2B). However, *SPP1* was present only in the malignant cells of tumors 1 and 4 (Figures S2B and S2C), suggesting that *SPP1* in malignant cells is not generally involved in TTR. In contrast, *MDK* and *POSTN* were prominently expressed in malignant cells at different time points of treatment in resistant tumors 1 and 2, while their expression was generally low in the responding tumor 3 (Figure S2C). Intriguingly, expression of *MDK* and *POSTN* increased with time in biopsies from tumor 4, correlating with the switch from therapeutic response to resistance formation in this patient (Figure S2C).

To confirm that high POSTN and MDK expression is associated with targeted therapy resistance, we performed an RNAscope assay for POSTN and MDK on melanoma tumors in a separate patient cohort (cohort 2, Figure 2C; Table S1) where patients were grouped according to fast progression versus slow progression disease after start of targeted therapy. To perform an unbiased statistical analysis, categorization of the patients in this cohort into fast vs. slow progression disease was done by determining the median progression-free survival (PFS) (136.5 days). The percentage of POSTN^+^ cells was significantly higher in four fast-progressing tumors compared to five slow-progressing tumors (*p* = 0.016, Figures 2D and 2E). Likewise, fast-progressing tumors displayed more prominent POSTN (also termed periostin) protein expression than slow-progressing tumors, as shown by immunohistochemical staining of tumor sections (Figure S2D). MDK expression was also notably higher in four fast-progressing tumors compared to four slow-progressing tumors (Figures 2F and 2G), although the difference was not significant (*p* = 0.19).

In various tumor types, *MDK* and *POSTN* have been found to be strongly expressed in cancer-associated fibroblasts (CAFs).^16^^,^^17^ Since our FNA technology failed to capture CAFs (Figure 1C), we investigated *POSTN* and *MDK* expression in the tumor microenvironment of targeted therapy-resistant melanoma biopsies using the Tumor Profiler dataset.^18^ In addition to their expression in therapy-resistant malignant cells, *POSTN* and *MDK* were expressed in several other cell populations, notably in fibroblasts of resistant tumors (Figures 2H and 2I). Thus, distinct cell types within a tumor can apparently act as a source for POSTN and MDK, raising the question about the identity of their effector cells.

### POSTN or MDK are unable to directly induce resistance against MEKi-driven cell death in melanoma cells

Potential effector cells of POSTN or MDK in resistant tumors could be the malignant cells themselves. To test whether POSTN or MDK could directly affect the responsiveness of melanoma cells to MAPK inhibitors, we first identified primary melanoma cell lines displaying either high or low endogenous POSTN and MDK expression (M150325, M131205 and M150543, M161201, respectively; Figures S3A and S3B). In the two POSTN^high^ cell lines, *POSTN* expression could be efficiently downregulated by transfection with POSTN siRNA (Figure S3C). However, both inhibition of POSTN in POSTN^high^ cell lines and reconstitution of POSTN levels in the POSTN^low^ cell lines by adding recombinant POSTN protein did not alter MEKi-induced pERK downregulation (Figures S3D and S3E). Accordingly, annexin V staining did not reveal a difference in the level of MEKi-induced cell death upon manipulation of POSTN levels (Figures S3F and S3G). Likewise, adding recombinant MDK to the MDK^low^ cell lines did also not stabilize pERK or increase cell survival upon MEKi treatment (Figure S3H). Thus, neither POSTN nor MDK altered the susceptibility of melanoma cells to MEKi-induced cell death.

### High expression of POSTN in resistant tumors is associated with infiltration of a specific macrophage population

**Figure 3:**
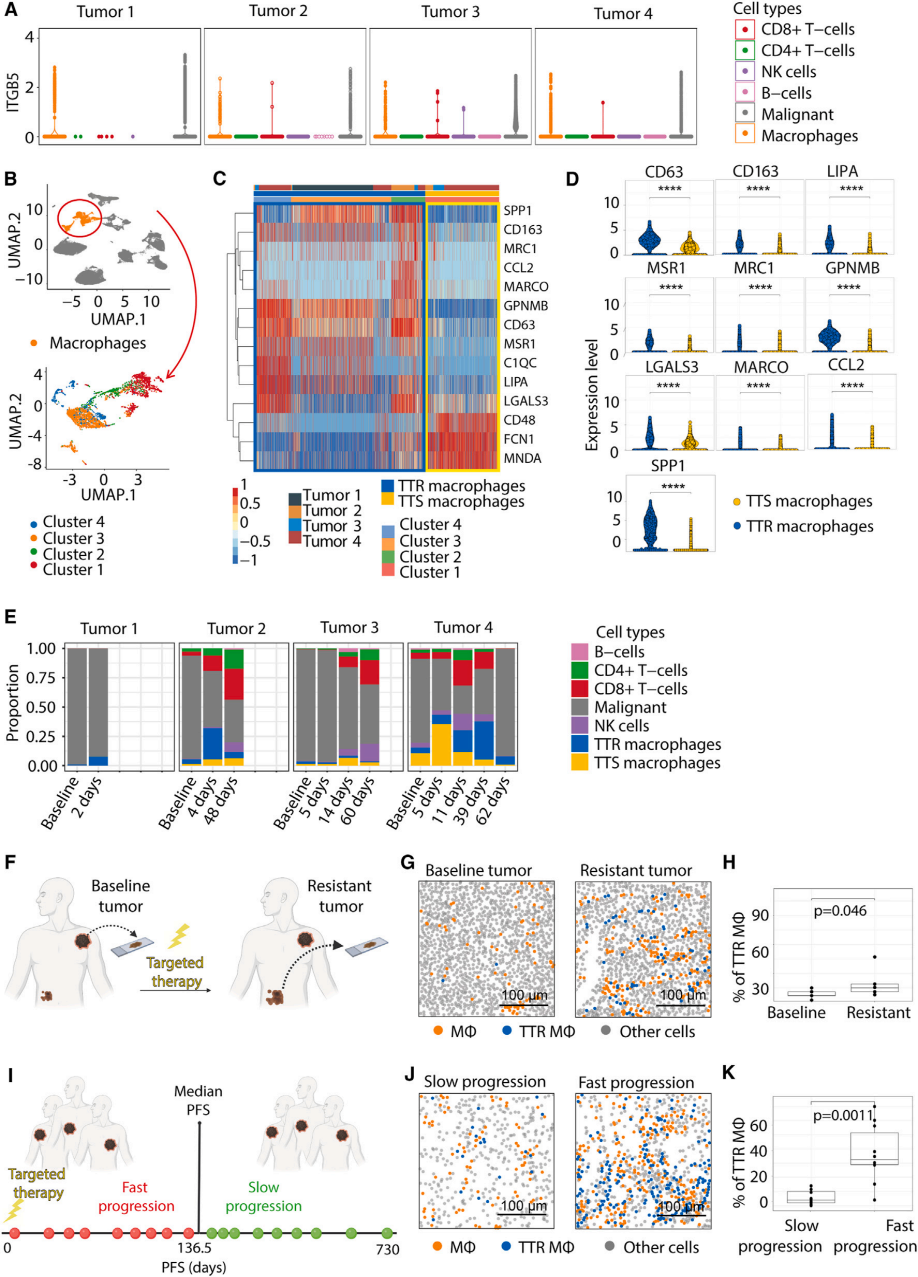
Resistant tumors are highly infiltrated with TTR macrophages (A) CellChat analysis of ITGB5 expression in cell populations between four tumors in cohort 1. (B) Upper UMAP plot showing total macrophage cluster in melanoma patients from cohort 1. The bottom UMAP plot highlights the segregation of macrophages into four populations. (C) Differential gene expression between the four macrophages populations. Cluster 1 represents TTS macrophages, and clusters 2, 3, and 4 are grouped into TTR macrophages. (D) Expression of 9 selected markers upregulated in TTR compared to TTS populations. (E) Cell-type proportion plots of identified cell populations including TTR and TTS macrophage for each tumor in cohort 1. (F) Schematic overview of cohort 3 (*n* = 4 patients). (G) TTR macrophage infiltration in melanoma tumors of cohort 3 at baseline and resistant stage. Scale bar 100 μm. (H) Quantification of TTR macrophages normalized to total macrophage numbers (*n* = 4 patients, *p* = 0.046, Wilcoxon rank-sum test). (I) Schematic overview of cohort 2 (*n* = 18 patients). (J) TTR macrophage infiltration in fast- and slow-progressing melanoma tumors of cohort 2: TTR macrophages (blue dots) and other macrophages (orange dots). Scale bar 100 μm. (K) Quantification of TTR macrophages between fast- and slow-progressing tumors (*n* = 9 tumors, *p* = 0.0011, Wilcoxon rank-sum test). MФ = macrophages.

As POSTN and MDK were unable to directly drive resistance in melanoma cells, we examined the cell populations that could be affected by POSTN or MDK. CellChat Explorer analysis of our scRNA-seq data from cohort 1 predicted that both POSTN and MDK mostly signal to macrophages, given the high expression of the POSTN receptors ITGB5, ITGAV, and ITGB3 (Figures 3A and S4A) and of several MDK receptors, such as LRP1 and NOTCH2 (Figure S4B), respectively, on these cells.

We then reclustered the macrophage population by unsupervised hierarchical clustering with the top 100 highly variable genes and found four distinct clusters (Figure 3B). Cluster 1 was notably different from other clusters and exhibited high expression of *CD48*, *MNDA*, and *FCN1* (Figure 3C), i.e., markers associated with a pro-inflammatory phenotype,^19^^,^^20^^,^^21^ suggesting that cluster 1 represented anti-tumorigenic macrophages likely associated with targeted therapy sensitivity (TTS). In contrast, clusters 2, 3, and 4 demonstrated common upregulation of several markers characteristic for a pro-tumorigenic phenotype, such as *CD63*, *GPNMB*, *MCR1*, *MSR1*, *CD163*, and *LIPA*^22^^,^^23^^,^^24^ (Figure 3C). Moreover, each of these clusters displayed high expression of a specific set of other phenotypic genes, such as *SPP1*, *CCL2*, *MARCO*, and *LGALS3* in cluster 2, *SPP1* and *C1QC*^19^^,^^25^ in cluster 3, and *LGALS3*, *MARCO*, and *C1QC* in cluster 4. Given their apparent pro-tumorigenic phenotype, we grouped clusters 2, 3, and 4 into one macrophage population predicted to represent (TTR)-associated macrophages. Among the markers significantly upregulated in TTR compared to TTS macrophages were *SPP1*, *CD63*, *CD163*, *LIPA*, *MSR1*, *MRC1*, *GPNMB*, *CCL2*, *MARCO*, and *LGALS3* (Figure 3D). Enrichment scores for both M1 and M2 macrophage signatures^26^^,^^27^ in TTR and TTS macrophages were low (<0.5), indicating that the TTS and TTR macrophage phenotypes do not belong to these classical categories (Figures S4C and S4D).

Cell-type proportion analysis in our tumors demonstrated that TTR macrophages were indeed present at high levels in resistant tumors 1 and 2 (Figure 3E). In contrast, in responding tumor 3, the proportion of TTR macrophages decreased, while the levels of TTS macrophages increased during treatment. In tumor 4, the proportion of TTR macrophages increased in accordance with the timeline of resistance formation, with predominant presence of TTR macrophages at late time points of treatment.

CellChat Explorer analysis using malignant cells as the sender and the TTR and TTS macrophage populations, respectively, as receivers, demonstrated that TTR macrophages expressed significantly higher levels of POSTN and MDK receptors compared to TTS macrophages (Figures S5A and S5B). Furthermore, CellChat Explorer analysis also predicted that the POSTN pathway was used by malignant cells to signal exclusively to TTR macrophages (Figure S5C).

Next, we validated the presence of TTR macrophages in a separate set of four melanoma patients (cohort 3) using paired biopsies collected before the start of BRAFi/MEKi therapy and at the resistant stage (Figure 3F; Table S1). Multiplex immunofluorescence using five specific TTR markers (CD163, GPNMB, LIPA, MSR1, and CD63) and the general macrophage marker CD68 identified one macrophage cluster (high CD68 and CD163), which could be further subdivided into three clusters, one with high CD68, one with high CD163, and one highly expressing all five TTR markers (Figures S5D–S5F). Consistent with the scRNA-seq data, these TTR macrophages were already present at the baseline (Figure 3G). Intriguingly, however, the proportion of TTR macrophages significantly increased at relapse (Figure 3H). To compare TTR macrophage infiltration between fast- and slow-progressing tumors from cohort 2 (Figure 3I), we performed an additional multiplex immunohistochemistry analysis and found a significant increase in the number of TTR macrophages in fast-progressing in comparison to slow-progressing tumors (Figure 3JJ and 3K). Taken together, resistance to BRAFi/MEKi in melanoma appears to be strongly correlated with increased TTR macrophage infiltration.

### POSTN expression polarizes macrophages toward a TTR phenotype and counteracts targeted therapy *in vivo*

**Figure 4:**
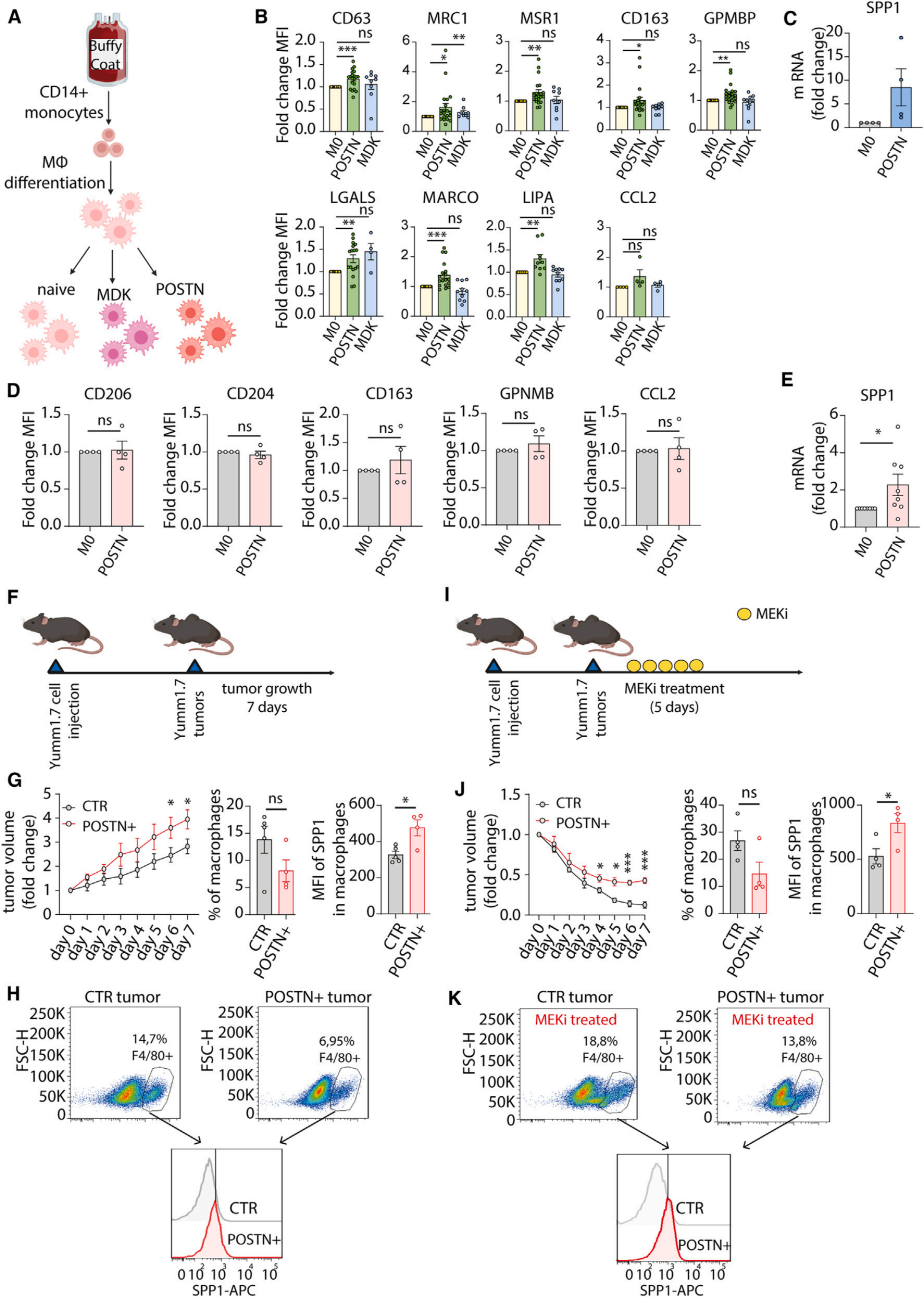
POSTN-polarized macrophages are associated with resistance of Yumm1.7 melanoma tumors to targeted therapy (A) Schematic overview of human macrophage differentiation and polarization. (B) Expression of TTR markers in human macrophages polarized with recPOSTN or recMDK. Flow cytometry data are shown as fold change of mean fluorescence intensity (MFI) normalized to non-polarized M0 macrophages (*n* ≥ 4 independent donors, two-tailed unpaired t test, mean ± SEM). (C) *SPP1* expression normalized to housekeeping gene *PPIA* between M0 and POSTN-polarized human macrophages (*n* = 4 macrophages from independent donors, unpaired t test, mean ± SEM). MФ = macrophages. (D) Expression of TTR markers in mouse macrophages polarized with recPOSTN. Flow cytometry data are shown as fold change of MFI normalized to non-polarized M0 macrophages (*n* = 4 independent mice, two-tailed unpaired t test, mean ± SEM). (E) *SPP1* expression normalized to housekeeping gene *GAPDH* between M0 and POSTN-polarized mouse macrophages (*n* = 8 macrophages from independent mice, unpaired t test, mean ± SEM). MФ = macrophages. (F) Illustration of experimental setup. BALB/c mice were subcutaneously inoculated with 10^6^ Yumm1.7-POSTN+ or Yumm1.7-CTR cells. 7 days after engraftment (day 0), the tumor volume was measured daily for 1 week (days 1–7). (G) Tumor growth representation of untreated tumors. Tumor volume was normalized as fold change to day 0 (*n* = 5 mice, two-way ANOVA test, mean ± SEM). Bar graphs show the percentage of F4/80+ macrophages from total cell population and MFI mean of SPP1 from the F4/80+ macrophage population (*n* = 5 tumors, unpaired t test, mean ± SEM). (H) Representative fluorescence-activated cell sorting (FACS) plots showing the F4/80+ macrophage population in the untreated tumors and SPP1 fluorescence intensity within the F4/80+ macrophage population. (I) Schematic representation of experimental setup. BALB/c mice were subcutaneously inoculated with 10^6^ Yumm1.7-POSTN+ and Yumm1.7-CTR cells. 7 days after engraftment (day 0), mice were treated daily with 1 mg/kg MEKi by peroral administration for 5 days. (J) Tumor growth representation of MEKi-treated tumors. Tumor volume was normalized as fold change to day 0 (*n* = 5 mice, two-way ANOVA test, mean ± SEM). Bar graphs show the percentage of F4/80+ macrophages from total cell population and MFI mean of SPP1 from the F4/80+ macrophage population (*n* = 5 tumors, unpaired t test, mean ± SEM). (K) Representative FACS plots showing the F4/80+ macrophage population in the MEKi-treated tumors and SPP1 fluorescence intensity within the F4/80+ macrophage population.

The results above raise the possibility that POSTN or MDK expression in melanoma mediate emergence of TTR macrophages and, as a consequence, resistance formation. To examine a potential association of these factors with the presence and function of TTR macrophages, we first assessed whether POSTN or MDK affects macrophage polarization. To this end, we differentiated macrophages from buffy coat-isolated monocytes and exposed them to recombinant POSTN or MDK for 3 days (Figure 4A), followed by expression analysis of the 10 markers used previously to characterize TTR macrophages on patient biopsies *in situ* (Figure 3D). Importantly, macrophages treated with POSTN displayed significant upregulation of most TTR macrophage markers, whereas MDK failed to polarize macrophages to a TTR phenotype (Figures 4B and 4C).

To address whether POSTN expression leads to presence of intratumoral TTR macrophages and resistance formation to targeted therapy *in vivo*, we sought to establish an immunocompetent melanoma mouse model system that is normally responsive to inhibition of the MAPK kinase pathway. There are well-known differences between mouse and human immune systems.^28^ Therefore, we first assessed whether POSTN could polarize mouse macrophages toward a phenotype reminiscent of human TTR macrophages. To this end, we isolated mouse bone marrow precursors, differentiated them toward macrophages, and polarized them with mouse recombinant POSTN for 3 days. However, none of the tested TTR markers, with the exception of SPP1, was upregulated in mouse macrophages after polarization (Figures 4D and 4E). Thus, mouse macrophages might not have the capacity to develop into TTR macrophages upon exposure to POSTN, or murine TTR macrophages might exhibit a somewhat distinct expression pattern compared to their human counterparts.

To probe the latter possibility, we performed *in vivo* experiments in a mouse melanoma model, using the Yumm1.7 cell line, which harbors the activating *Braf*^*V600E*^ mutation, is homozygous negative for *Pten* and *Cdkn2*, and is syngeneic with immunocompetent C57/B1/6 mice.^29^^,^^30^ Yumm1.7 demonstrated high susceptibility to MEKi treatment (Figure S5G). Using lentiviral vectors, we created a Yumm1.7 cell line overexpressing mouse *POSTN* (Yumm1.7 POSTN^+^) and a control cell line infected with empty vector (Yumm1.7 CTR) (Figure S5H). Both created cell lines showed comparable sensitivity to MEKi treatment, suggesting that enhanced expression of POSTN did not influence cell susceptibility to targeted therapy (Figure S5I).

To examine if overexpression of POSTN could promote the emergence of SPP1+ macrophages also *in vivo*, both Yumm1.7- POSTN^+^ and Yumm1.7-CTR cell lines were subcutaneously inoculated in mice. The tumor volumes were measured daily for 7 days before euthanizing the mice. Intriguingly, Yumm1.7- POSTN^+^ tumors were growing notably faster than control tumors. Furthermore, flow cytometry analysis of macrophage infiltrates in tumors at endpoint revealed that although the total number of macrophages was not significantly different between both conditions, macrophages in POSTN^+^ tumors expressed significantly higher SPP1 level (Figures 4G and 4H). These data indicate that high POSTN expression in tumors polarize tumor-infiltrating mouse macrophages into an SPP1-high phenotype.

Next, we investigated whether POSTN overexpression in tumors could confer resistance to targeted therapy. Yumm1.7- POSTN^+^ and Yumm1.7-CTR cell lines were subcutaneously inoculated in mice and, 1 week later (day 0), POSTN^+^ and CTR tumor-bearing mice were treated with MEKi for 5 days. Tumor sizes were monitored daily, and 7 days after therapy start (day 7), mice were euthanized, and tumor-infiltrating macrophages were analyzed by flow cytometry (Figure 4I). The total number of macrophages was slightly decreased in POSTN^+^ tumors; however, these macrophages expressed significantly higher SPP1 levels (Figures 4J and 4K). Importantly, POSTN^+^ tumor volumes of MEKi-treated mice were considerably larger than in the control. In summary, overexpression of POSTN in melanoma led to a high number of intratumoral SPP1+ macrophages, which was associated with a significantly reduced response to targeted therapy.

### TTR macrophages cannot be used as predictive markers in baseline tumors

**Figure 5:**
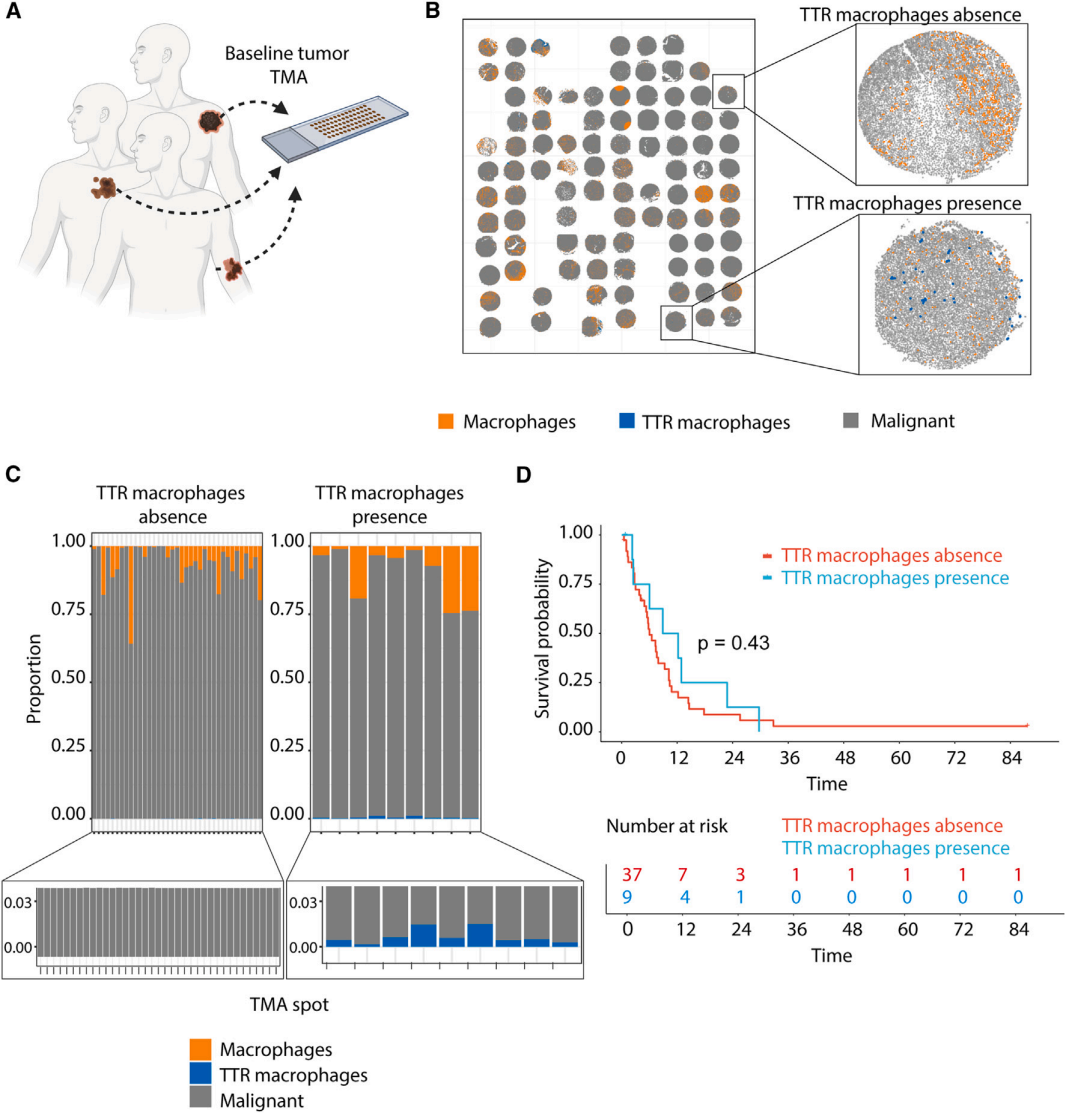
Presence of TTR macrophages cannot be used as predictive marker for targeted therapy response (A) Schematic overview of cohort 4. Biopsies for tissue microarray (TMA) were taken from 46 patients before (baseline) targeted therapy was applied. The cohort includes patients who responded to the treatment as well as patients who developed resistance during the treatment. (B) Representative digital picture of TMA multiplex immunohistochemistry staining indicating TTR macrophages, other macrophages, and malignant cells in baseline tumors of patients in cohort 4: TTR macrophages (blue dots) and other macrophages (orange dots). Malignant cells are indicated as gray dots. TMA slots are vertically duplicated. (C) Graph showing TTR macrophage-positive and TTR macrophage-negative baseline tumors of patients in cohort 4 and proportional quantification of TTR macrophages, other macrophages, and malignant cells in these tumors. One bar represents one TMA slot or one patient (*n* = 46 patients). (D) Kaplan-Meier survival curves for patients in cohort 4 who were positive for TTR macrophages in baseline tumors (*n* = 9 patients) or negative for TTR macrophages in baseline tumors (*n* = 37 patients). Log rank test *p* = 0.43.

To assess whether TTR macrophages at baseline could be used as a predictive marker for response, we used a tissue microarray (TMA) of a melanoma cohort of baseline tumors from patients who responded or acquired resistance after BRAFi/MEKi treatment (cohort 4, Figure 5A; Table S1). We performed a multiplex immunohistochemistry using five specific TTR markers (Figure 5B). Nine of the patient biopsies had TTR macrophages ranging from 0.1%–14% of all cells, whereas no TTR macrophages were detectable in 37 patient samples (Figure 5C). Survival analysis of these two groups revealed no differences in PFS (Figure 5D). These data suggest that TTR macrophages cannot be used as a predictive marker for response to targeted therapy but could potentially be used as a biomarker during treatment.

### TTR macrophages are predominant in targeted therapy

**Figure 6:**
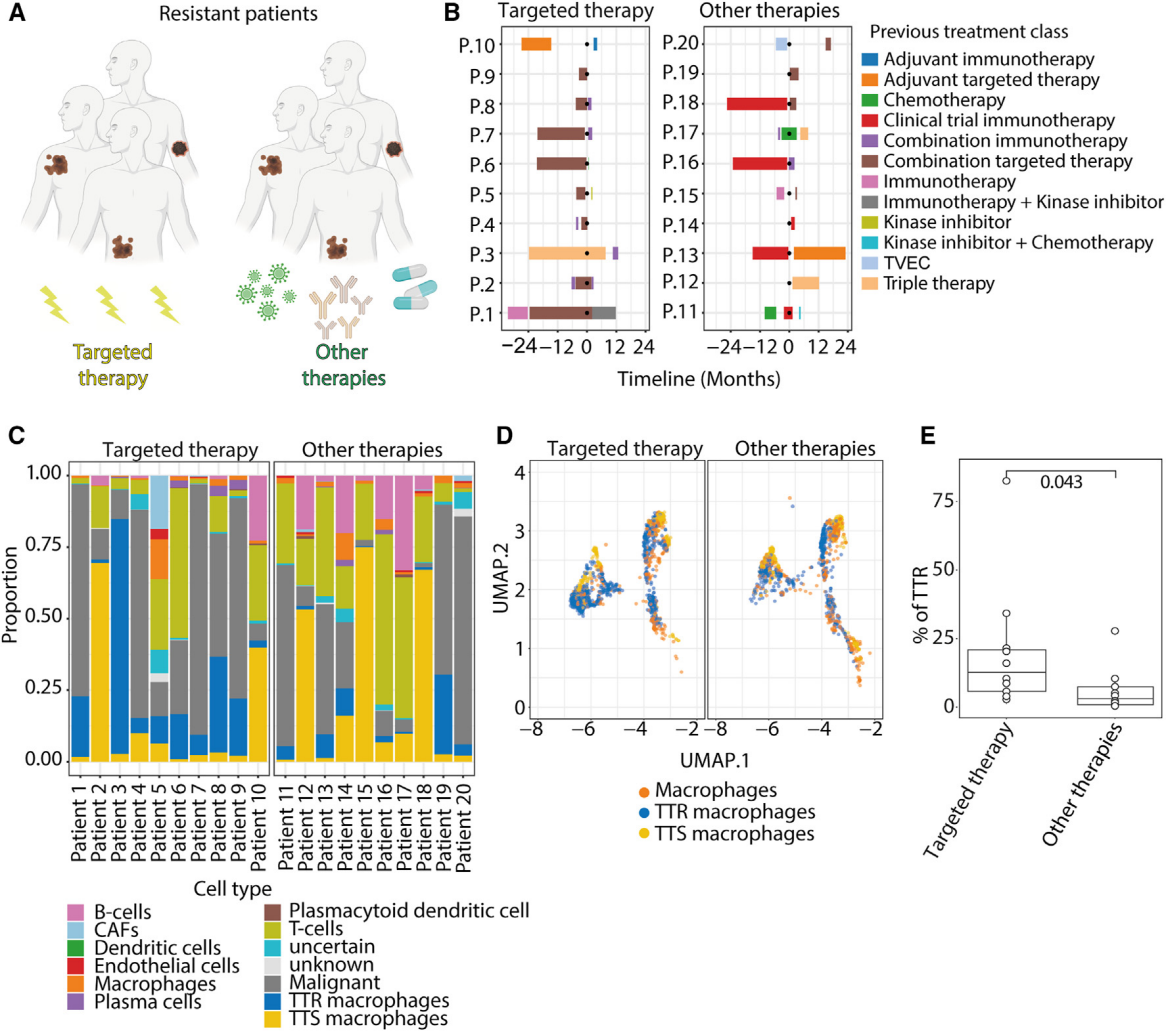
TTR macrophages predominantly infiltrate tumors resistant to BRAFi/MEKi therapy (A) Schematic overview of cohort 5 (*n* = 20 patients). (B) Swimmer’s plots showing the two subgroups of patients in cohort 5 that were either treated with targeted therapy or other therapies. The timeline and applied therapies are indicated. (C) Cell-type proportion plots of identified cell populations obtained from all biopsies of patients in cohort 5. (D) UMAP plot of macrophages colored by subtype showing the proportion of macrophage subtypes in patients of cohort 5. Macrophages that had both TTS and TTR macrophage enrichment scores less than or higher than the median were labeled as “macrophages,” macrophages with a TTR macrophage enrichment score higher than the median were labeled as “TTR macrophages,” and macrophages with a TTS macrophage enrichment score higher than the median were labeled as “TTS macrophages.” (E) Proportional quantification of TTR macrophages between resistant patients treated with targeted therapy and other therapies (*n* = 20 melanoma tumors, *p* = 0.043, Wilcoxon rank-sum test).

To investigate whether TTR macrophages were specific for BRAFi/MEKi resistance, we interrogated the Tumor Profiler scRNA-seq dataset.^18^ We analyzed 10 resistant biopsies from patients during or after BRAFi/MEKi treatment and 10 resistant biopsies from patients treated with another type of therapy (cohort 5, Figures 6A and 6B; Table S1). In contrast to cohort 1, cell proportion analysis of cohort 5 showed a broader cellular composition of the biopsies where, in addition to malignant cells and immune cells, endothelial cells and CAFs were detected (Figure 6C). AddModuleScore was used to determine the enrichment scores of the TTR and TTS macrophage signatures for the macrophages in this dataset (Figure 6D). The percentage of TTR macrophages was significantly higher in the samples treated with BRAFi/MEKi compared to samples treated with another type of therapy (Figure 6E). This suggests that TTR macrophages are predominant for resistance to BRAFi/MEKi treatment.

### POSTN-polarized TTR macrophages confer resistance to targeted therapy in melanoma cells *in vitro*

**Figure 7:**
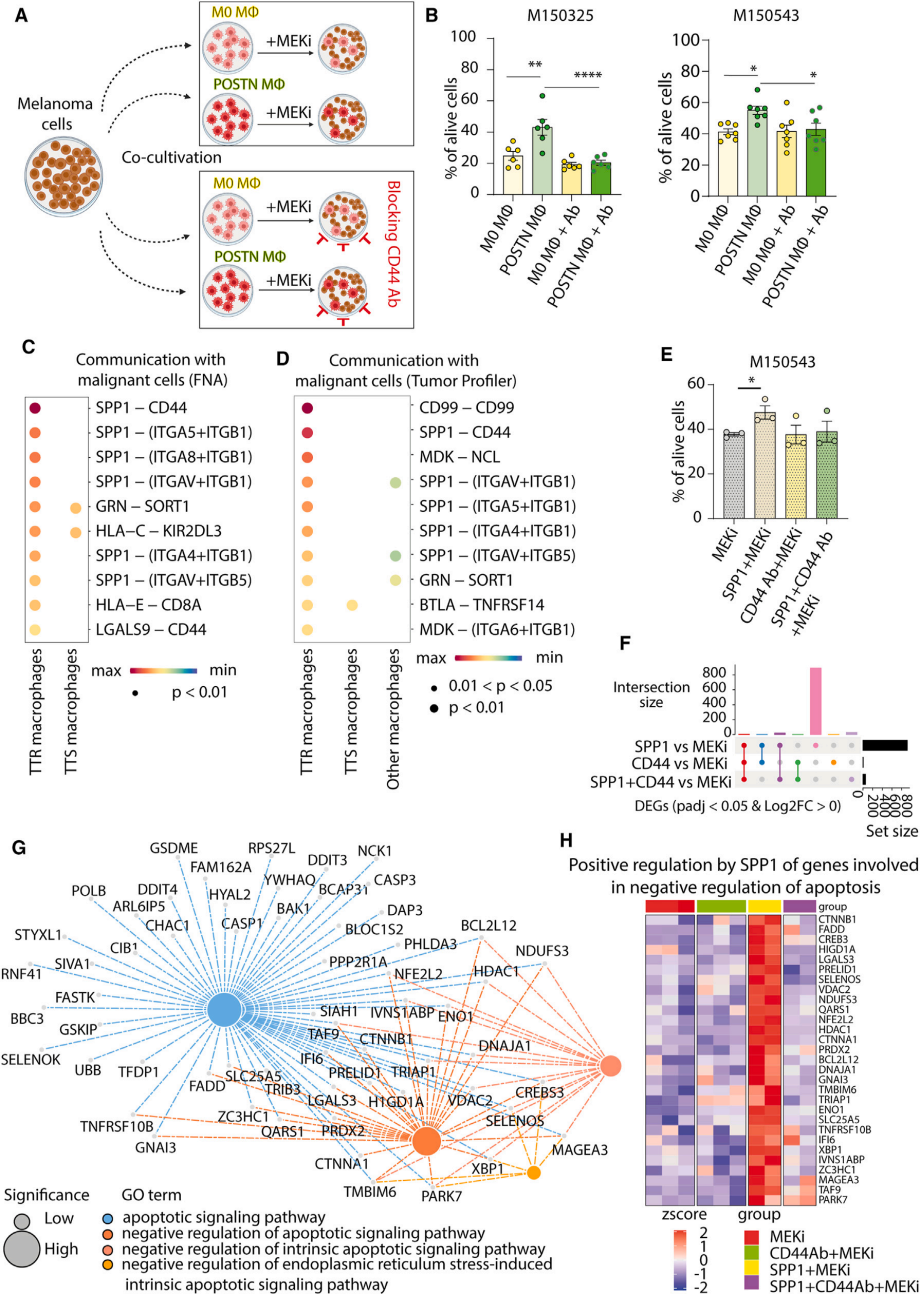
SPP1-CD44 is one of the resistance-driving mechanisms in melanoma cells (A) Schematic representation of co-cultivation experiment. Non-polarized M0 or POSTN-polarized TTR macrophages were cultured with melanoma cells and treated with MEKi for 4 days (upper panel). Melanoma cells were co-cultured with TTR or M0 macrophages, pretreated with CD44 blocking antibody and then treated with MEKi for 4 days (bottom panel). (B) Quantification of alive melanoma cells after co-cultivation with TTR macrophages and 4-day treatment with MEKi in presence of CD44 blocking antibodies (Ab) (*n* = 6–7 macrophages from independent donors, ordinary one-way ANOVA test, mean ± SEM). (C) Bubble plot showing ligand-receptor-based CellChat analysis of the FNA scRNA-seq dataset from cohort 1, with TTR and TTS macrophages as senders and the malignant cells as receivers. (D) Bubble plot showing ligand-receptor-based CellChat analysis of cohort 5 with the TTR, TTS, and other macrophages as senders and the malignant cells as receivers. MФ = macrophages. (E) Quantification of alive melanoma cells after 2 h pre-treatment with SPP1- or CD44-blocking antibodies (Ab) or combined SPP1+CD44-blocking antibody followed by a 3-day treatment with MEKi (*n* = 3 independent experiments, ordinary one-way ANOVA test, mean ± SEM). (F) UpSet plot for the differentially upregulated genes (*p* adjusted <0.05, log2 fold change ˃ 0) between the distinct treatments versus MEKi in the cell line M150543. M150543 cells were treated with MEKi for 20 h following a 2-h pre-stimulation with recombinant SPP1- or CD44-blocking antibody or combined recombinant SPP1+ CD44-blocking antibody. (G) Network plot for representative apoptosis-related GO terms linked to the uniquely upregulated genes in SPP1+MEKi treatment condition (pink vertical bar in F). Nodes are color-coded based on the specific GO terms. Size of the nodes map the comparative level of significance (all GO terms enriched with *p* adjusted <0.05). Colored dotted lines connect the genes with the respective GO terms they belong to. (H) Heatmap depicting the expression of genes positively regulated by SPP1 included in the GO terms “negative regulation of apoptotic signaling pathway.”

Our results showed a strong association between presence of intratumoral TTR macrophages and melanoma resistance formation in mice and patients upon targeted therapy. To investigate whether polarized TTR macrophages could directly influence the response of melanoma cells to MEKi treatment, we cultured two MEKi-sensitive cell lines (M150325 and M150543) with either naive macrophages or POSTN-induced TTR macrophages, followed by treatment with MEKi for 4 days (Figure 7A upper panel). The annexin V cell death assay showed that POSTN-induced TTR macrophages significantly prevented MEKi-induced cell death in melanoma cells, in contrast to naive macrophages (Figure 7B).

To determine a mechanism by which TTR macrophages could drive resistance formation in melanoma, we first examined factors known to mediate this process, such as VEGF^31^ and TNFα.^32^ However, these factors were expressed at low levels in TTR and TTS macrophages (Figure S6A). Next, we applied CellChat analysis to our FNA scRNA-seq datasets (cohort 1), with the TTR and TTS macrophages as senders and the malignant cells as receivers. There were 104 and 106 ligand-receptor pairs for TTS and TTR macrophages with malignant cells, respectively (Figure S6B). Analysis of ligand-receptor pairs with highest crosstalk prediction for TTR macrophages revealed SPP1 as one of the most active ligands involved in communication via several integrin receptors and with highest probability via CD44 receptor (Figure 7C). To validate our findings on a patient level, we performed similar CellChat Explorer analysis on the Tumor Profiler dataset^18^ (cohort 5), annotating the TTS, TTR, and other macrophages as senders and the malignant cells as receivers. There were 55, 12, and 42 ligand-receptor pairs for TTR macrophages, TTS macrophages, and other macrophages, respectively (Figure S6C). Again, SPP1 was identified as a distinctive ligand for TTR macrophages communicating via several receptors, where CD44 again demonstrated the highest communication probability (Figure 7D). The data suggest that SPP1-CD44 may represent one of the most likely mechanisms of crosstalk between TTR macrophages and malignant cells in human melanoma. Supporting these data, expression analysis of *SPP1* across all cell types demonstrated that *SPP1* was predominantly expressed in TTR macrophages (Figure S7A). Expression of *CD44* receptors was found at high levels in malignant cells and other cell types (Figure S7B).

To study whether CD44 might mediate TTR macrophage-induced resistance formation, we first treated two melanoma cell lines exhibiting high CD44 expression with CD44 blocking antibodies^33^ in the presence or absence of MEKi. However, blocking CD44 activity in the absence of exogenous activating ligands did not alter the susceptibility of melanoma cells to MEKi treatment (Figures S7C and S7D). In contrast, when melanoma cells pretreated with CD44 blocking antibodies were mixed before MEKi treatment with either differentiated TTR or naive macrophages (Figure 7A, bottom panel), CD44 blockade was able to reverse TTR macrophage-mediated protection of MEKi-induced cell death (Figure 7B). These results indicate that TTR macrophages mediate the development of resistance in melanoma cells via CD44 downstream pathways.

To investigate whether SPP1 is the main ligand responsible for resistance formation via CD44 signaling, we conducted *in vitro* experiments, in which the MEKi-sensitive cell lines M150325 and M150543 were treated with recombinant SPP1, both with and without the presence of a CD44 blocking antibody, followed by treatment with MEKi. Our analysis showed that activation of CD44 signaling with SPP1 ligand increased the survival of M150543 cells upon MEKi treatment (Figure 7E). However, the protection against cell death provided by SPP1 treatment in M150543 cells was comparatively weaker than that observed upon co-cultivation with TTR macrophages. Notably, SPP1 treatment failed to protect M150325 cells from MEKi-induced death, suggesting that TTR macrophages secrete alternative factors that also activate CD44 signaling (Figure S7E). Taken together, our data demonstrate that, while SPP1 can induce resistance, the secretome released by TTR macrophages contains additional factors that contribute to resistance formation via CD44 signaling.

Focusing on the resistance mechanism mediated by SPP1-CD44 signaling, we first investigated whether previously reported canonical CD44 signaling pathways^34^^,^^35^^,^^36^ could be involved in resistance formation in M150543 cells. M150543 cells were subject to MEKi alone or the following combinatorial treatments: SPP1+MEKi; CD44 blocking antibody +MEKi; or SPP1+CD44 blocking antibody+MEKi. However, flow cytometry analysis of pERK, pAKT, pSRC, pSTAT3, pVEGFR, pEGFR, and pERBB2 and the transcription factors NANOG, OCT4, SOD2, and SOX2 did not reveal any significant differences in the expression levels of these proteins under our treatment conditions (Figure S7G). Therefore, we performed bulk RNA sequencing analysis of M150543 cells treated as above. Of note, SPP1+MEKi treatment had a strong effect on the transcriptional profile of M150543 cells, separating this condition from the other experimental groups along the first principal component (Figure S7F). Interestingly, differential gene expression analysis demonstrated the presence of a large number of specific upregulated genes (*p* adj < 0.05) upon SPP1+MEKi treatment (Figure 7F). Functional analysis of these SPP1+MEKi-treament-specific genes retrieved significant enrichment for Gene Ontology (GO) terms linked to regulation of apoptosis (Figure 7G), including distinct annotations related to the negative regulation of apoptosis. Moreover, genes included in the GO terms “negative regulation of apoptosis” showed both a specific and strong expression pattern in the SPP1+MEKi-treated group compared to other treatments (Figure 7H). These findings support our hypothesis that SPP1 may represent an important mediator of TTR and point to the blockade of apoptotic pathways as a potential mechanism.

## Discussion

The most limiting factor for the long-term use of targeted therapy in melanoma is the development of acquired resistance that typically evolves after 6–12 months of treatment. To unravel the mechanisms mediating resistance formation, we used repeated FNA-based tumor sampling for longitudinal monitoring of patient response combined with scRNA sequencing. The in-depth analysis of 14 FNA samples in responding and resistant tumors revealed a major difference in the melanoma cell secretome. Unlike therapy-responding tumors, resistant tumors were found to express high levels of POSTN, which based on computational analysis mediates the communication with a macrophage subpopulation (TTR macrophages) predominantly present in patients’ biopsies resistant to targeted therapy as opposed to targeted therapy-sensitive tumors or tumors resistant to other types of therapies. Macrophages exposed to POSTN adopted a TTR phenotype, which was associated with escape from targeted therapy-induced death in malignant cells *in vitro* and in melanoma *in vivo*. This protective effect appeared to be mediated through CD44 signaling, with SPP1 identified as a primary ligand responsible for the interaction between TTR macrophages and malignant cells in tumors resistant to targeted therapy.

Identification of resistance-associated cellular phenotypes and characterization of their signatures have been the focus of many research studies over the past decades. Sampling by FNA, followed by scRNA-seq, allows monitoring these processes in minimally manipulated tumor biopsies from patients on treatment. However, although we were able to observe changes in malignant and immune cells at different time points during treatment, there are some limitations associated with the FNA technique. For instance, we failed to capture some stromal cell populations, like CAFs, by FNA, leaving open the contribution of these cells to the establishment of treatment resistance. By comparing malignant cells of our patients with available datasets, we detected some of the previously described phenotypes, such as “proliferative,”^14^^,^^15^ “melanocytic,”^10^ “pigmented,”^9^ or “MITF-high.”^8^ Of note, however, cell stages previously related to resistance, such as “invasive,”^9^^,^^14^^,^^15^ “undifferentiated,” “undifferentiated neural crest-like,” “neural crest-like,”^10^ and “AXL-high,”^8^ were not found. The absence of these populations in our dataset could be explained by patient-specific differences in tumor composition and activation of alternative mechanisms of resistance to targeted therapy. In addition, many of these resistance-associated cellular phenotypes were identified in cell cultures or PDX mouse models, where loss of the human tumor stroma and absence of the proper immune cell composition could influence the phenotype of malignant cells.

In our study, we show a strong correlation between high expression of POSTN in melanoma cells and resistance formation, where the source of POSTN could be malignant cells and stromal cells, such as CAFs. POSTN has been previously implicated in resistance formation in different cancers in response to a variety of therapeutic approaches. For instance, in mouse xenograft models of human glioma, high POSTN mediated enhanced expression of TGFB1 and HIF1 alpha, which resulted in acquired resistance to anti-VEGF-A therapy.^37^ In another study, gene expression profiling of ovarian patients identified POSTN as one of the top three highest ranked signature genes associated with clinical chemoresistance.^38^ Moreover, POSTN was shown to have a direct effect on the response of malignant cells to drugs *in vitro*. For example, high POSTN induced resistance formation to cisplatin via activation of the AKT pathway in ovarian adenocarcinoma^39^ and non-small cell lung cancer (NSCLC) cells.^40^ Likewise, overexpression of POSTN in a human gastric carcinoma cell line resulted in decreased 5-fluorouracil-induced apoptosis.^40^ Furthermore, POSTN was able to promote resistance to carboplatin and paclitaxel in ovarian cancer cells.^38^ Liu et al. showed that high POSTN activated AKT and stabilized MAPK signaling in BRAF-mutant A375 melanoma cells to bypass BRAFi/MEKi-induced death.^41^ In our study, however, treatment with recombinant POSTN neither resulted in stabilization of the MAPK pathway nor increased survival of melanoma cells upon MEKi treatment, suggesting that POSTN plays an indirect effect on resistance formation. In line with this hypothesis, POSTN was shown to be a potent pro-tumorigenic immunomodulator predominantly affecting TAM recruitment, for instance in glioblastoma xenografts where silencing of POSTN in tumor cells led to reduced infiltration of M2-like TAMs, inhibited tumor growth, and prolonged mouse survival.^42^^,^^43^ Similarly, high levels of POSTN in ascetic fluids of ovarian cancer patients correlated with high CD163^+^ TAM recruitment and with decreased relapse-free survival. Our study reveals a key role for POSTN in the tumor microenvironment in polarizing macrophages toward a pro-tumorigenic phenotype. In this context, POSTN has been poorly investigated. POSTN was shown to increase chemotaxis in THP1 monocytes and to upregulate CD206 (MRC1) in THP1 macrophages, indicating M2 polarization.^44^ In culture experiments using human buffy coat-derived macrophages, we show that POSTN induced expression of a specific set of markers (SPP1, CD163, CD63, LIPA, MSR1, MRC1, GPNMB, MARCO, and LGALS3) also present on TTR macrophages found in human melanoma biopsies. Despite the known differences between mouse and human immune systems,^28^ one of these markers, SPP1, was also induced by POSTN both in murine macrophages in culture and in intratumoral macrophages in a mouse melanoma model. This was associated with increased tumor growth and, importantly, with significantly reduced response to targeted therapy *in vivo.*

Combinations of our TTR macrophage signature have previously been reported in humans to identify TAMs with specific phenotypic features. For instance, Wang et al. demonstrated that CD163^+^ GPNMB^+^ macrophages induce a pro-invasive phenotype in melanoma cells.^45^ LIPA was reported to contribute to pro-tumorigenic education of TAMs.^22^^,^^23^^,^^24^ MARCO-expressing TAMs were shown to accumulate in NSCLC tissue, to be associated with pro-tumorigenic activity, and to counteract the activity and tumor cell-killing ability of cytotoxic T cells and NK cells in culture.^19^^,^^25^ Furthermore, single-cell profiling of myeloid cells from patients with breast cancer identified lipid-associated macrophages that co-expressed the TTR macrophage markers CD63, LIPA, GPNMB, MCR1, CD163, and MSR1. Depletion of these macrophages in TREM*2* KO mice was associated with delayed tumor growth, indicating the tumor-promoting role of these macrophages in the tumor microenvironment.^44^ Finally, mapping of the glioblastoma immune landscape in mouse tumors and in patients using scRNA-seq and CITE-seq identified macrophages co-expressing GPNMB, LGALS3, FABP5, and CD63.^46^ We also identified TTR macrophages in an independent dataset from the Tumor Profiler consortium where TTR macrophages were detected predominantly in melanoma patients resistant to targeted therapy but much less in patients treated with other therapies, suggesting that these macrophages are specific for TTR.

Previous evidence supports the notion that macrophages could trigger resistance in BRAF-mutant melanoma. Targeted therapy led to high infiltration of TNFα-expressing TAMs in BRAF-mutant melanoma patients and in melanoma allografts.^32^ Moreover, TNFα from THP1 macrophages induced expression of the transcription factor MITF in melanoma cells, which was associated with a decrease in BRAFi/MEKi-induced cell death. Consequently, suppression of TNFα-signaling in melanoma allografts enhanced the efficacy of targeted therapy.^32^ In another study, treatment with BRAFi led to MAPK signaling activation and high VEGF production in macrophages that increased melanoma cell survival upon BRAFi treatment in co-culture assays.^31^ However, neither of these resistance-driving mechanisms were detected in our analysis. In our co-culture experiments, melanoma cells were efficiently killed by MEKi treatment despite the presence of macrophages, unless the latter where pre-exposed to POSTN, indicating that POSTN-polarized TTR macrophages mediate resistance formation against targeted therapy in melanoma. Given these findings and the reported roles of POSTN in cancer progression, targeting POSTN might appear as an attractive therapeutic approach. Indeed, in preclinical breast^47^^,^^48^^,^^49^ and ovarian^50^ cancer models, blockade of POSTN revealed good therapeutic results. However, POSTN is implicated in a large number of physiological and pathological processes, and there is a broad list of cell types that express POSTN, suggesting that anti-POSTN therapies could induce multiple unwanted systemic side effects in patients.

Therefore, to discover other potential treatment options for targeted therapy-resistant melanoma patients, we investigated communication mechanisms possibly mediating the protective effect of TTR macrophages on drug-exposed malignant cells. In human patients, TTR but not TTS or other macrophages expressed high levels of the secreted factor SPP1 that was predicted to signal to CD44^+^ malignant cells. SPP1^+^ macrophages have been associated with cancer progression in glioblastoma^24^ and colorectal cancer^19^ and resistance formation to chemotherapy in lung adenocarcinoma.^51^ Furthermore, the SPP1-CD44 communication pathway between macrophages and cancer cells has been reported to accelerate malignant progression of glioma^52^ and gastric cancers.^53^ In our study, the tumor cell protection ability of POSTN-polarized TTR macrophages was greatly suppressed in the presence of CD44-neutralizing antibodies, demonstrating a role for this pathway in targeted therapy resistance formation in melanoma. SPP1 turned out to be one of the ligands contributing to CD44-mediated resistance formation. Mechanistically, we found that stimulation of the SPP1-CD44 pathway appears to protect melanoma cells from targeted therapy-induced cell death by upregulation of genes inhibiting apoptosis. However, SPP1 treatment offered less protection against cell death compared to co-cultivation with TTR macrophages and failed to prevent MEKi-induced death in all cell lines, demonstrating that resistance formation by TTR macrophages is also driven by alternative factors. CD44 is heavily expressed in many types of tumors and is apparently involved in tumor aggressiveness and metastatic potential.^54^^,^^55^ A phase I clinical study applying the anti-CD44 antibody Bivatuzumab in cancer patients demonstrated drug tolerance, although clinical efficacy of the therapy was low, resulting in stable disease in only 21% of the patients.^56^ However, our study indicates that CD44 might represent a promising therapeutic target in combination with targeted therapy in melanoma where anti-CD44 antibodies or other agents blocking CD44-mediated cellular crosstalk could suppress TTR macrophage-driven resistance formation and, hence, could improve therapeutic outcome.

### Limitations of the study

One limitation of our study is the low number of patients in the clinical cohort 1. Therefore, our strategy for employing FNA was to gain initial insights into specific mechanisms of TTR to be validated in additional patient cohorts. However, a larger number of patients and more extensive FNA sampling may have provided more accurate assessments of the prevalence and function of TTR macrophages upon targeted therapy.

Secondly, by using a syngeneic mouse melanoma model, we confirmed the involvement of TTR macrophages in resistance formation to targeted therapy. However, POSTN-polarized mouse macrophages lack many of the characteristics of human TTR macrophages. Utilizing humanized mouse models would be better suited to model the functional contribution of TTR macrophages to resistance formation *in vivo*.

Thirdly, out of several ligand-receptor communication pathways between TTR macrophages and malignant cells, we validated only the most prominent one, SPP1-CD44. It is plausible that multiple factors secreted by TTR macrophages induce resistance development, possibly in a synergistic manner, warranting further investigation in future studies.

## STAR★Methods

### Key resources table

**Table:** 

REAGENT or RESOURCE	SOURCE	IDENTIFIER
**Antibodies**		

Rabbit anti-human CD68 (EPR20545) antibody	Abcam	Cat# ab213363; RRID:AB_2801637
Rabbit anti-human CD163 antibody	Abcam	Cat# ab182422; RRID:AB_2753196
Rabbit anti-human CD204 (EPR24403-17) antibody	Abcam	Cat# ab271070
Rabbit anti-human LIPA antibody	Novus Biologicals	Cat# NBP1-54155; RRID:AB_11028324
Mouse anti-human CD63 (MX-49.129.5) antibody	Santa Cruz	Cat# sc-5275; RRID:AB_627877
Rat anti-human CD44 (Hermes-1) antibody	ThermoFisher Scientific	Cat# MA4400; RRID:AB_223517
Mouse anti-human GPNMB (D-9) antibody	Santa Cruz	Cat# sc-271415; RRID:AB_10610660
Goat anti-human POSTN (S-15) antibody	Santa Cruz	Cat# sc-49480; RRID:AB_2166653
Mouse anti-human POSTN (F-10)	Santa Cruz	Cat# sc-398631; RRID:AB_2934053
Human BD Fc (Fc1) Block	BD Biosciences	Cat# 564219; RRID:AB_2728082
Anti-human CD163-Violet 605 (GHI/61) antibody	BioLegend	Cat# 333616; RRID:AB_2616879
Anti-human CD206-BUV395 (19.2) antibody	BD OptiBuild	Cat# 740309; RRID:AB_2740047
Anti-human CD204-PE-Cyanine 7 (7C9C20) antibody	BioLegend	Cat# 371908; RRID:AB_2650772
Anti-human CD63-APC-Cyanine 7 (H5C6) antibody	BioLegend	Cat# 353046; RRID:AB_2860923
Anti-human MARCO-APC (PLK-1) antibody	Invitrogen	Cat# 17-5447-42; RRID:AB_2762440
Anti-human CD68-Violet 421 (Y1/82A) antibody	BioLegend	Cat# 333828; RRID:AB_2800882
Anti-human MCP1-APC (5D3-F7) antibody	BioLegend	Cat# 502612; RRID:AB_2734489
Anti-human Galectin-PE (GAL397) antibody	BioLegend	Cat# 126706; RRID:AB_2075197
Anti-human CD68-PE (Y1/82A) antibody	BioLegend	Cat# 333808; RRID:AB_1089056
Anti-human CD44-FITC (MEM-263) antibody	Novus Biologicals	Cat# NBP1-42789; RRID:AB_2074683
Anit-mouse F4/80-FITC antibody	Invitrogen	Cat# 11-4801-82; RRID:AB_2637191
Anti-mouse GPNMB-eFluor 660 antibody	Invitrogen	Cat# 50-5708-82; RRID:AB_2574239
Anti-mouse CD204-PE Cyanine 7 antibody	Invitrogen	Cat# 25-2046-82; RRID:AB_2637412
Anti-mouse CD206-Alexa fluor 700 antibody	Invitrogen	Cat# 56-2061-82; RRID:AB_2762723
Anti-mouse CD163-eFluor 450	Invitrogen	Cat# 48-1631-82; RRID:AB_2815180
Anti-CCL2-PE	Invitrogen	Cat# 12-7096-82; RRID:AB_466171
Rabbit anti-human,mouse SPP1 antibody	ThermoFisher Scientific	Cat# PA5-141129; RRID:AB_2932581
Anti-Annexin V-APC antibody	BioLegend	Cat# 640941
Donkey anti-Goat Alexa Fluor® 488 antibody	Jackson	Cat# 705-545-147; RRID:AB_2336933
Goat anti-Rabbit APC antibody	Invitrogen	Cat# A10931; RRID:AB_2534068
Goat anti-Mouse IgG1 Alexa Fluor 488 antibody	Invitrogen	Cat# A-21121; RRID:AB_2535764
Goat Anti-Rabbit Cy™3 IgG antibody	Jackson	Cat# 111-165-003; RRID:AB_2338000
Mouse anti-human α-Tubulin (B-5-1-2) antibody	Sigma-Aldrich	Cat# T-6074; RRID:AB_477582
Mouse anti-human total p44/42 MAPK (L34F12) antibody	Cell Signaling	Cat# 4696; RRID:AB_390780
Rabbit anti-human hosphor-p44/42 MAPK antibody	Cell Signaling	Cat# 9101; RRID:AB_331646
Donkey anti-rabbit IRDye 680LT secondary antibody	LI-COR Biosciences	Cat# 926–68023; RRID:AB_10706167
Donkey anti-mouse IRDye 800CW secondary antibody	LI-COR Biosciences	Cat# 926–32212; RRID:AB_621847

**Biological samples**		

FNA biopsies	This paper	N/A
FFPE melanoma tumor sections	URPP Biobank	N/A
Tumor Profiler melanoma biopsies	Irmisch et al.^18^	N/A
Buffy coats	Zurich blood donation services	N/A

**Chemicals, peptides, and recombinant protein**		

7AAD	BD Biosciences	51-68981E
Dapi	Sigma-Aldrich	D9542
DMSO	Sigma Aldrich	D4540
FBS (fetal bovine serum)	Biowest	S-181H-500
Endotoxin low Qualified FBS	Gibco	10270–106
Human recombinant M-CSF	PeproTech	300–25
Mouse recombinant M-CSF	PeproTech	315–02
Human recombinant MDK	PeproTech	450–16
Human recombinant POSTN	R&D systems	3548-F2-050
Mouse recombinant POSTN	R&D systems	2955-F2-050
Human recombinant SPP1	Abcam	ab281819
L-Glutamine	ThermoFisher Scientific	A2916801
Penicillin-Streptomycin	ThermoFisher Scientific	1,5E+07
Trametinib	Active Biochemicals	A-1258
Liberase	Roche	05401054001
DNase I	Roche	10104159001
Avidin-biotin alkaline phosphatase complexes	Vector laboratories	AK-5002
Vector® Red Substrate Kit, Alkaline Phosphatase	Vector laboratories	AK-5100
LightCycler 480 SYBR Green I Master	Roche	4,7E+09

**Critical commercial assays**		

Chromium Next GEM Automated Single Cell 3′ cDNA Kit v3.1	10X GENOMICS	1000424
Chromium Next GEM Automated Single Cell 3′ Library and Gel Bead Kit v3.1	10X GENOMICS	1000147
Chromium Next GEM Chip G Single Cell Kit	10X GENOMICS	1000127
Opal Polaris 7-Color Automated IHC Detection Kit	Akoya Biosciences	NEL871001KT
RNAscope Probe Hs-POSTN-C3	ACD	409181-C3
RNAscope Probe - Hs-MDK-O1	Bio-Techne	586471
EasySep™ Human CD14 Positive Selection Kit II	STEMCELL	17858
JetPrime transfection kit	Polyplus transfection	114
Direct-zol RNA MiniPrep	Lucerna-Chem	R2050
High Capacity cDNA Reverse Transcription Kit	Applied Biosystems	4368814
Flow Cytometry Fixation and Permeabilization Buffer Kit I	R&D Systems	FC009
BCA Protein Assay Kit	Thermo Fisher Scientific	23227
DuoSet ELISA kits for POSTN	R&D systems	DY3548B
DuoSet ELISA kits for MDK	R&D systems	DY258
TruSeq Stranded mRNA	Illumina, Inc	N/A

**Deposited data**		

FNA single-cell RNA-seq data	GEO accession number	GEO: GSE229908
The code of FNA single-cell RNA-seq data	N/A	Zenodo: https://doi.org/10.5281/zenodo.11150125
FNA single-cell RNA-seq data analysis and the processed Seurat object	N/A	Zenodo: https://doi.org/10.5281/zenodo.10930890
Bulk-sequencing data	GEO accession number	GEO: GSE262779
The code of the bulk RNAseq data	N/A	Zenodo: https://zenodo.org/doi/10.5281/zenodo.11174282
The Tumor Profiler data	N/A	Zenodo: https://zenodo.org/records/11208953
The code to generate the figures associated with the Tumor Profiler	N/A	Zenodo: https://zenodo.org/records/11209193

**Experimental models: Cell lines**		

M131205 melanoma cell line	URPP biobank	N/A
M150325 melanoma cell line	URPP biobank	N/A
M161201 melanoma cell line	URPP biobank	N/A
M150543 melanoma cell line	URPP biobank	N/A
Yumm1.7 mouse melanoma cell line	ATCC	CRL-3362 ™

**Experimental models: Organisms/strains**		

Mouse C57BL/6	The Jackson Laboratory	JAX:000664

**Oligonucleotides**		

POSTN Silencer® Select siRNA	ThermoFisher Scientific	4392420
Silencer® Select Negative Control #1 siRNA	ThermoFisher Scientific	4390843
qRT-PCR primers, see Table S2	This paper	N/A

**Recombinant DNA**		

pLV-EGFP-EF1A-mPostn	VectorBuilder	VB231128-1117paq
pLV-EGFP	VectorBuilder	N/A

**Software and algorithms**		

RTCA Software 2.1.0	ACEA Biosciences	S2807-90004
FlowJo 7.6	Software.informre	https://flowjo.software.informer.com/7.6/
ImageJ V 1.8.0 software	softonic	https://imagej.en.softonic.com/download
GraphPad Prism 5.0	GraphPad Software. Inc.	https://www.graphpad.com/
Cellranger 3.0.1	10X genomics	https://support.10xgenomics.com/single-cell-gene-expression/software/downloads/latest
Phenochart 1.1.0	Akoya Biosciences	https://www.akoyabio.com/support/software/phenochart-whole-slide-viewer/
InForm 2.6.0	Akoya Biosciences	https://www.akoyabio.com/support/software/inform-tissue-finder-software/
Discovery Workbench 4.0	Mesoscale	https://www.mesoscale.com/en/products_and_services/software#
R 4.2.0	R project	https://cran.r-project.org/mirrors.html
Giotto (version 2.0.0.957)	R package	https://github.com/drieslab/Giotto/tree/suite
Seurat	R package	https://satijalab.org/seurat/index.html
scDblFinder	R package	https://doi.org/10.18129/B9.bioc.scDblFinder
SingleR	R package	https://doi.org/10.18129/B9.bioc.SingleR
ggplot2	R package	https://ggplot2.tidyverse.org/index.html
harmony	R package	https://github.com/immunogenomics/harmony
infercnv	R package	https://github.com/broadinstitute/infercnv
Cellchat	R package	https://github.com/sqjin/CellChat
FastQC (version 0.11.9)	Babraham Bioinformatics	https://www.bioinformatics.babraham.ac.uk/projects/fastqc/
Trimmomatic (version 0.39)	USADELLAB.org	https://github.com/usadellab/Trimmomatic
STAR (version 2.7.10a)	Dobin et al.^57^	https://github.com/alexdobin/STAR
R (version 4.0.2)	R project	https://cran.r-project.org/mirrors.html
DESeq2 (version 1.28.1)	Bioconductor	https://github.com/thelovelab/DESeq2
ClusterProfiler (version 3.16.1)	Bioconductor	https://bioconductor.org/packages/release/bioc/html/clusterProfiler.html
ComplexHeatmap (version 2.4.3)	Bioconductor	https://github.com/jokergoo/ComplexHeatmap
igraph (version 1.3.5)	CRAN	https://CRAN.R-project.org/package=igraph

**Other**		

Histopaque-1077	Sigma-Aldrich	10771-500M
DMEM + GlutaMAX medium	Gibco	21885–025
RPMI 1640 medium	ThermoFisher Scientific	1,2E+07
Annexin V buffer	BD Bioscience	51-66121E
RIPA buffer	Thermo Fisher Scientific	89901
Odyssey blocking buffer	LI-COR Biosciences	927–40000
Target Retrieval Solution, Citrate pH 6	DAKO	S2369
Hematoxylin	Sigma-Aldrich	109254
VectaMount™ Mounting Medium	Vector laboratories	H-5000
Tween™ 20 Surfact-Amps™ Detergent Solution	Thermo Fisher Scientific	5113

### Resource availability

#### Lead contact

Further information and requests for resources and reagents should be directed to and will be fulfilled by the lead contact, Lukas Sommer (lukas.sommer@anatomy.uzh.ch).

#### Materials availability

This study did not generate new unique reagents.

#### Data and code availability

The Tumor Profiler data are accessible through Zenodo: https://zenodo.org/records/11208953. The code to generate the figures associated with the Tumor Profiler data are accessible through Zenodo: https://zenodo.org/records/11209193.

Newly generated transcriptomic datasets reported in this paper have been deposited in NCBI’s Gene Expression Omnibus. Processed scRNA sequencing files are accessible through GEO series accession number GEO: GSE229908. The code is provided in Zenodo https://doi.org/10.5281/zenodo.11150125 and the processed Seurat object at Zenodo: https://doi.org/10.5281/zenodo.10930890.

Bulk RNA sequencing datasets are accessible through GEO series accession number GEO: GSE262779. The code used for the analyzing the bulk RNAseq data and generating the respective figures included in the manuscript is available at https://github.com/adsalas/SPP1_in_melanoma_resistance and has been deposited in Zenodo with the Zenodo: https://doi.org/10.5281/zenodo.11174283 (https://zenodo.org/doi/10.5281/zenodo.11174282).

Any additional information required to reanalyze the data reported in this work paper is available from the lead contact upon request.

### Experimental model and study participant details

#### Patient cohorts and buffy coats

Cohort 1. Four patients with metastatic melanoma who were planned to be treated with the combination of BRAF and MEK inhibitors (BRAFi/MEKi), had cutaneous or subcutaneous metastases and have provided informed consent, were eligible for the fine needle aspiration (FNA, (BASEC# 2018-00379)). We collected FNA from selected tumors (melanoma metastases) before treatment start, within the first week of BRAFi/MEKi treatment and at following regular clinical visits for as long tumors were accessible and patients have consented. As the majority of the melanoma patients show clinical response to BRAFi/MEKi, we classified tumors in the FNA cohort based on the best response observed until the last known assessment. Tumors were classified as responding, if the tumor has completely disappeared during the BRAFi/MEKi therapy, or resistant, if the tumor has never completely disappeared, even if it shrunk during the treatment course. Tumors that have disappeared completely and recurred later have been classified corresponding to their status at the time of assessment.

Cohort 2. Fast and slow progressing tumors. Formalin fixed paraffin embedded (FFPE) samples were collected from 18 consenting patients with metastatic melanoma treated with BRAFi/MEKi (FFPE cohort, KEK-ZH-Nr. 647, 800, BASEC: 2017-00494, BASEC: 2014.0425). As the majority of the melanoma patients show clinical response to BRAFi/MEKi, but develop resistance and disease progression during treatment course, a clinically relevant outcome is progression free survival (PFS), which defines the time from treatment start and first documented disease progression. In the cohort 2, the median PFS was 136.5 days. We calculated the median PFS for the whole cohort and classified patients as fast progressors (PFS shorter than the median PFS of the whole cohort) and slow progressors (PFS longer than median of the cohort).

Cohort 3. Baseline and resistant tumors from the same patient. In this cohort, we analyzed paired samples from four consenting patients treated with BRAFi (KEK-ZH-Nr. 647, 800, BASEC: 2017-00494, BASEC: 2014.0425). FFPE samples were collected within 2 months before the start of BRAFi and within 30 days after progression (detected by PET-CT). Baseline and resistant tumor samples were collected from the same patient but not from the same tumor.

Cohort 4. Melanoma BRAFi/MEKi tissue microarray analysis (TMA). The TMA contains 46 FFPE baseline melanoma tumors collected within two months before the start of BRAFi (*n* = 17) or BRAFi/MEKi (*n* = 29) from consented patients (KEK-ZH-Nr. 647, 800, BASEC: 2017-00494, BASEC: 2014.0425). Each tumor had two representative cores on the TMA. 29 patients were treatment naive, 16 were previously treated with immunotherapy and one with chemotherapy. Median PFS was 6 months (95% CI 4.9–10.2) in the TTR absent cohort and 10.6 months (95% CI 6 – NA) in the TTR present cohort.

Cohort 5. Tumor Profiler patients. The Tumor Profiler cohort (BASEC-2018-02050) includes single cell RNAseq data from 20 resistant melanoma tumors. 10 tumor biopsies were collected from patients during or after BRAFi/MEKi treatment and 10 tumor biopsies after another type of therapy (immunotherapy, TVEC, or chemotherapy).

Buffy coats were obtained from anonymized healthy adult donors provided by the Zurich blood donation services (ZHBDS) (BASEC-NR: Req-2021-00661). All experiments involving samples from human donors were conducted with the approval of the ethics committee of Canton of Zurich, Switzerland.

#### Melanoma cell cultures

Primary human melanoma cell cultures M131205, M150325, M161201 and M150543 were established and provided by the URPP biobank at the Department of Dermatology, University Hospital Zurich. Primary melanoma cell cultures were generated from excess tumor material of surgically removed melanoma metastases from patients after written informed consent and approved by the local IRB (BASEC: 2017-00494, BASEC: 2014.0425). Clinical diagnosis of tumor material was confirmed by histology and immunohistochemistry.

Human melanoma cells were cultured in RPMI 1640 supplemented with 10% heat-inactivated FBS, 4 mM L-Glutamine and Penicillin-Streptomycin (complete RPMI) in a humidified incubator at 37°C and 5% CO2. All cell lines were tested for mycoplasma by a PCR-based method and were found to be negative.

Yumm1.7 mouse malignant melanoma cell line was purchased from American Type Cell Culture (ATCC) and cultured in DMEM supplemented with 10% heat inactivated FBS in a humidified incubator at 37°C and 5% CO2. The cell line was tested for mycoplasma by a PCR-based method and was found to be negative.

#### Animal experimental models

8 week-old C57BL/6 female mice were purchased from The Jackson laboratory. Animals were housed in a certified animal facility with a 12-h light/dark cycle, with free access to water and food and at temperatures of 21°C–23°C and humidity of 40–60%. All animal experiments were approved by the veterinary office of Canton of Zurich, Switzerland and were performed in accordance with Swiss law (ZH013/2022). Modified Yumm1.7 CTR and Yumm1.7 POSTN+ cells were harvested by trypsinization and resuspended in PBS before injection to animals. 10^6^ cells in 50 μL PBS were injected subcutaneously into the back of mice. During inoculation, mice were kept under inhalational anesthesia with isoflurane. One week after tumor cell inoculation the experiments with tumor measurements and targeted therapy administration were started.

### Methods details

#### FNA sample collection

The collected FNA samples were resuspended in a freezing medium consisting of 10% DMSO, 90% fetal bovine serum (FBS) and slow-frozen in freezing container Mr. Frosty (Nalgene) as described.^58^ The FNA collection was approved by the local ethics committee (BASEC# 2018-00379).

#### Single cell sequencing analysis

Raw sequencing data were processed using the 10× Chromium Cell Ranger pipeline (version 3.0.1) (https://support.10xgenomics.com/single-cell-gene-expression/software/downloads). Reads were aligned to the human reference genome (GRCh38, 2018) (10x Genomics). Seurat v3 pipeline using SCTransform was used to normalize gene expression data. Data integration was performed using 3000 features and PrepSCTIntegration.^59^ Dimension reduction was performed using Principal Component Analysis (PCA). Clustering was performed using the Louvain algorithm with a resolution of 0.5. Projection onto two-dimensional space was performed with Uniform Manifold Approximation and Projection (UMAP) using 30 dimensions. Single cells were filtered with the following parameters: >500 genes and <9000 genes detected, <40% mitochondrial RNA, and defined as “singlet” by scDblFinder.^60^ Manual cell typing of clusters was performed using the FindAllMarkers function in Seurat. The macrophage clusters were determined by taking the top 100 highly variable genes and performing unsupervised hierarchical clustering. The dendrogram presented four major clusters which were used for downstream analysis. InferCNV was used to estimate copy number variation of all single cells.^61^ Malignant cells were determined by unsupervised hierarchical clustering for a cluster having specific CNV alterations. Melanoma phenotype scoring was calculated using the AddModuleScore function from the Seurat package from the following signatures Hoek,^14^ Verfaillie,^15^ Tirosh,^8^ Tsoi,^10^ and Rambow.^9^ Differential expression was performed using the find Markers function from the scran package.^62^ ggplot2 was used for visualizing aggregated data.^63^ Ligand receptor interactions were evaluated using CellChat Explorer.^64^ We used all cell types as senders and receivers; macrophages were split into TTS and TTR macrophages. Tumor Profiler single-cell data were generated in the context of an approved clinical study (BASEC: 2018–02050).

#### Multiplex immunofluorescence

FFPE samples from Cohorts 3, 4 and 5 were stained. BOND RXm fully automated staining system was used in conjunction with the Opal Polaris 7-Color Automated IHC Detection Kit according to the manufacturer' instructions. Slides were imaged simultaneously using the Akoya Vectra Polaris imaging system maintained by the Center for Microscopy and Image Analysis, University of Zurich (Zurich, Switzerland).

Slide visualization and regions of interest selection were performed in Phenochart (v1.1.0) whole-slide viewer (Akoya Biosciences). Whole-slide scans can be made available on reasonable request. Inform software (version 2.6) was used for cell segmentation. 4′,6-diamidino-2-phenylindole (DAPI) nuclear staining was used for nucleus detection, followed by cell marker phenotyping (CD68, CD163, MSR1, LIPA, GPNMB, CD63). In-depth spatial expression analysis was performed using Giotto (version 2.0.0.957).^65^ Briefly, cytoplasmic fluorescence signals were normalized. Dimension reduction was performed with PCA and then UMAP. Clustering was performed with Leiden clustering using a resolution of 0.1. Clusters were manually annotated based on marker protein expression.

#### RNAscope

Formalinfixed paraffinembedded (FFPE) tumor sections from consenting melanoma patients were provided by URPP Biobank, Department of Dermatology, University Hospital Zurich. Section deparaffinization, antigen retrieval, and staining were performed according to RNAscope Multiplex Fluorescent Reagent Kit v2 Assay protocol. To stain POSTN or MDK-expressing cells the RNAscope Probe Hs-POSTN-C3 or RNAscope Probe Hs-MDK-O1 were applied respectively at dilution 1:50. Nuclei were counterstained with DAPI (dilution 1:2000). Melanoma sections were imaged using the Akoya Vectra Polaris imaging system maintained by the Center for Microscopy and Image Analysis, University of Zurich (Zurich, Switzerland). Slide visualization and regions of interest selection were performed in Phenochart (v1.1.0) whole-slide viewer (Akoya Biosciences).

#### IHC staining

IHC was performed on formalin-fixed paraffin-embedded tumor specimens using avidin-biotin alkaline phosphatase complexes according to the manufacturer’s protocol. After deparaffinization, an antigen-retrieval step was performed using Target Retrieval Solution, Citrate pH 6 for 45 min in a Microwave Histoprocessor. The sections were incubated for 1 h at room temperature with Periostin primary antibody (dilution 1:50) and Vector red was used as substrate. The sections were counterstained using Hematoxylin for 3 min. Subsequently, the sections were washed and mounted with VectaMount Mounting Medium.

#### Human macrophage differentiation

Buffy coats were diluted in PBS (1:1) and peripheral blood mononuclear cells (PBMCs) were isolated by density gradient centrifugation using Histopaque-1077. Monocytes were purified using EasySep Human CD14 Positive Selection Kit II and immunomagnetic column-free EasySep magnet (STEMCELL, 18000) according to the manufacturer’s guidelines. Isolated monocytes were cultured in complete macrophages medium DMEM + GlutaMAX containing 10% endotoxin low qualified FBS and recombinant human M-CSF (50 ng/mL) for 7 days. Matured, differentiated macrophages were used for migration assays or re-plated for polarization experiments.

#### Human macrophages polarization

After differentiation, matured macrophages were detached using PBS containing 2 mM EDTA for 20 min at 37°C. Cells were seeded in complete macrophage medium (without recombinant human M-CSF) at a density of 10^5^ cells per well of a 48-well plate. Cells were polarized by stimulation with recombinant human MDK (100 ng/mL) or recombinant human POSTN (100 ng/mL) for 3 days. Non-stimulated macrophages were used as a negative control.

#### Mouse macrophage differentiation and polarization

Bone marrow precursors were flushed from long bones of C57BL/6 and cultured in DMEM supplemented with 10% endotoxin low qualified FBS, in the presence of 10 ng/ml of M-CSF. At day 4 non-adherent cells were collected and cultured for a further 3 days in the presence of fresh media. On day 7, the media was replaced with complete fresh media containing recombinant mouse POSTN (100 ng/mL) for 3 days. At day 10 cells were harvested and analyzed by flow cytometry and qRT-PCR.

#### Treatment of tumor-bearing mice and tumor processing

One week after inoculation of Yumm1.7 CTR and Yumm1.7 POSTN+ tumor cells, when the xenografts reached around 200 mm^3^+/−50 mm^3^, mice were randomized into 2 groups for each cell line. One group of CTR and POSTN+ tumor-bearing mice were kept in untreated conditions with a daily monitoring of tumor growth for 7 days. Second group of CTR and POSTN+ tumor-bearing mice were treated via daily peroral administration of the Trametinib (1 mg/kg) and tumor growth was daily monitored. After 5 doses of daily treatment, therapy was interrupted and mice were kept without treatment for 2 more days. The tumor growth of melanoma xenografts was calculated by formula: (W/2×W/2×L/2)×3/4×π, where W-width, L-length and π = 3,14.At the experimental endpoint the mice were euthanized in a CO_2_ chamber and tumors were processed. The tumors were first cut and chopped into small pieces and then incubated in digestion buffer (RPMI medium containing liberase (13 units/ml) and DNAse (0.1 mg/mL)) in an orbital shaker for 45 min at 37°C. The reaction was stopped by adding RPMI supplemented with 10% FBS. To get better single cell suspension the cell mix was resuspended using syringe and 26G needle and filtered in 40 μm cell strainer. The mix was centrifuged at 1200 rpm for 10 min at RT and cell pellet was fixed for flow cytometry analysis.

#### Cell co-cultivation experiment

M150325 and M150543 melanoma cells were cultured as described above. Naive or POSTN-polarized TTR macrophages were obtained as described above. Melanoma cells and macrophages were counted and mixed at a 3:1 ratio. This cell mix was seeded on a 48-well plate at density 10^5^ cells for M150543 and 2×10^5^ cells for M150325 cells. For the cell mix containing TTR macrophages, recombinant POSTN was added at a final concentration 100 ng/mL. Cell mix containing naive macrophages were cultured without additional cytokines. 24 h after plating, cells were treated with 50nM Trametinib for 96 h. In experiments using CD44 blocking antibody, cell mix was pretreated with CD44 monoclonal antibody (Hermes-1) (dilution 10 μg/mL) for 2 h before the start of MEKi treatment. Cell death was analyzed using Annexin V cell death assay by Flow cytometry. Expression of CD44 was analyzed by Flow cytometry.

#### siRNA transfection and treatment with MEKi

Cells were cultured in complete growth medium and transfected at 60% confluency using a final concentration of 50 pmol POSTN Silencer Select siRNA. As a control a Silencer Select Negative Control siRNA has been used. For transfection, the JetPrime transfection kit was used according to the manufacturer’s guidelines. In a case of MEK treatment, 24 h after transfection, cells were incubated with 50nM of Trametinib for 72 h. Expression of *POSTN* was analyzed by qRT-PCR.

#### Cell treatment with recPOSTN/recMDK/recSPP1 and MEKi

Cells were cultured in complete growth medium. Reaching the 60% confluence, cells were stimulated with recombinant human POSTN or recombinant human MDK at a final concentration of 500 ng/mL for 24 h. On the next day after stimulation, cells were treated with 50 nM of Trametinib for 72 h. Upon drug treatment, fresh recombinant POSTN and MDK was added every day to the cells.

Stimulation with recombinant human SPP1 (500 ng/mL) or CD44 blocking antibodies (10 μg/mL) was performed 2 h before MEKi treatment. For cell survival experiments, 50 nM MEKi treatment was applied for 72 h. For analysis of proteins downstream of CD44 signaling, cells were cultured in starvation medium (Complete RPMI medium containing only 1% of FBS) and 50 nM MEKi was applied for 6 h.

#### Bulk RNA sequencing

M150543 cells were cultured in starvation medium (complete RPMI medium containing only 1% FBS). Reaching 80% confluence, cells were stimulated with recombinant human SPP1 (500 ng/mL) or CD44 blocking antibodies (10 μg/mL) for 2 h. In combinatorial treatment, first, CD44 receptor was blocked with CD44 blocking antibodies for 2 h before signaling pathway activation by recombinant SPP1 for 2 h. 50 nM MEKi treatment was applied for 20 h. RNA was collected using the Direct-zol RNA MiniPrep according to manufacturer’s guidelines. The quality of the isolated RNA was determined with a Fragment Analyzer. The TruSeq Stranded mRNA was used in the succeeding steps. Briefly, total RNA samples (100–1000 ng) were poly A enriched and then reverse-transcribed into double-stranded cDNA. The cDNA samples were fragmented, end-repaired and adenylated before ligation of TruSeq adapters containing unique dual indices (UDI) for multiplexing. Fragments containing TruSeq adapters on both ends were selectively enriched with PCR. The quality and quantity of the enriched libraries were validated using the Fragment Analyzer. The product is a smear with an average fragment size of approximately 260 bp. The libraries were normalized to 10nM in Tris-Cl 10 mM, pH8.5 with 0.1% Tween 20.Sequencing configuration was paired-end 150 bp. The Novaseq 6000 was used for cluster generation and sequencing according to standard protocol. Fastq files were checked for quality with FastQC (version 0.11.9) and trimmed using Trimmomatic (version 0.39).Trimmed fastq files were aligned against the human genome annotation (GRCh38 release 105, retrieved from Ensembl) using STAR algorithm (version2.7.10a).^57^ Count tables were imported into R (version 4.0.2) for further processing. Differential gene expression analysis was performed with DESeq2 (version 1.28.1)^66^ using as reference the MEKi treated group. DEGs with a p adjusted value <0.05 were considered significantly changing. Functional analysis of biological processes was performed with ClusterProfiler (version 3.16.1)^67^ using as input the uniquely up-regulated genes (log2 fold change ˃ 0) retrieved for the SPP1 treatment. Heatmap and network representations of specific biological processes were made using the packages ComplexHeatmap (version 2.4.3) and igraph (version 1.3.5), respectively.

#### RNA isolation and qRT-RCR

RNA extraction and DNase treatment were performed using the Direct-zol RNA MiniPrep according to manufacturer’s guidelines. Purified RNA was quantified by nanodrop and subjected to reverse transcriptase reaction using High Capacity cDNA Reverse Transcription Kit according to manufacturer’s recommendations. Real-time quantitative PCR (qRT-PCR) was performed on a LightCycler 480 System (Roche) using LightCycler 480 SYBR Green I Master. The qRT-PCR primers are listed in Table S2. Relative quantified mRNA was normalized for human to housekeeping *PPIA* gene transcripts and for mouse to housekeeping *GAPDH* gene transcripts.

#### Flow cytometry

For assessment of melanoma cell death, cells were harvested, washed with Annexin V buffer and stained with Annexin V-APC (dilution 1:100) and 7AAD (dilution 1:100) according to the manufacturer’s BD Bioscience instructions. For POSTN expression, melanoma cells were harvested, stained with FC block, then fixed and permeabilized with Flow Cytometry Fixation and Permeabilization Buffer Kit I following manufacturer’s recommendations. Cells were stained with primary antibody anti-POSTN (dilution 1:100) for 30 min at 4C and with secondary Donkey anti-Goat Alexa Fluor 488 (dilution 1:1000) for 30 min at 4°C.

To evaluate TTR signatures on macrophages, the cells were treated with human FC block, fixed and permeabilized with Flow Cytometry Fixation and Permeabilization Buffer Kit I according to manufacturer’s guidelines. Then macrophages were stained with anti-Galectin-PE (dilution 1:200), anti-MCP1-APC (dilution 1:100), anti-CD68-Violet 421 (dilution 1:200), anti-MARCO-APC (dilution 1:200), anti-CD63-APC-Cyanine 7 (dilution 1:200), anti-CD204-PE-Cyanine 7 (dilution 1:200), anti-CD206-BUV395 (dilution 1:200), anti-CD163-Violet 605 (dilution 1:200), anti-Periostin (dilution 1:100), anti-GPNMB (dilution 1:100), anti-LIPA (dilution 1:200) for 30 min at 4°C degrees. For uncoupled antibodies we additionally stained the cells with secondary Donkey anti-Goat Alexa Fluor 488 (dilution 1:1000), Goat anti-Mouse IgG1 Alexa Fluor 488 (dilution 1:500) and Goat Anti-Rabbit Cy3 IgG (dilution 1:1000).

For mouse macrophages differentiated *in vitro*, cells were fixed and permeabilized with Flow Cytometry Fixation and Permeabilization Buffer Kit I according to manufacturer’s guidelines. Then macrophages were stained with anti-F4/80-FITC (dilution 1:100) anti-GPNMB-eFluor 660 (dilution 1:100), anti-CD204-PE-Cyanine 7 (dilution 1:100), anti-CD206-Alexa fluor 700 (dilution 1:100), anti-CD163-eFluor 450 (dilution 1:100) and anti-CCL2-PE (dilution 1:100) for 30 min at 4°C degrees.

For the analysis of Yumm1.7 tumor infiltrating macrophages, tumor and tumor infiltrating cells were fixed and permeabilized with Flow Cytometry Fixation and Permeabilization Buffer Kit I according to manufacturer’s guidelines. Next cells were stained with anti-F4/80-FITC (dilution 1:100) and anit-SPP1 (dilution 1:50) for 30 min at 4°C degrees. Then cells were stained with goat anti-rabbit APC secondary antibodies (dilution 1:500) for 30 min at 4°C degrees.

For the co-culturing experiment, to distinguish cancer cells from macrophages, the cell mix was harvested, blocked with human FC block for 15 min and stained with anti-CD68-PE (dilution 1:200) for 30 min. The cell mix was then washed with Annexin V buffer and stained with Annexin V-APC (dilution 1:100) and 7AAD (dilution 1:100) following the manufacturer’s BD Bioscience guidelines.

Expression of CD44 receptor was analyzed using CD44 antibody (dilution 1:100).

The samples were analyzed on a BD FACS Canto II or BD FACSymphony flow cytometer. FlowJo 7.6 was used to process the obtained data.

#### Protein isolation and western blotting

Cultured cells were lysed with RIPA buffer containing Phophatse Inhibitor Coctail and sonicated using Bandelin SONOPULS ultrasonic homogenizer (Merck) for 10 s with pulsation power of 20%. Protein concentrations were determined using the BCA Protein Assay Kit and quantified using a DTX 880 Multimode Detector at 562 nm. Protein samples were supplemented with 4x Laemmli Sample Buffer (Bio-Rad) containing 10% 2-mercaptoethanol and denaturated at 37°C for 30 min 80 μg of total protein per sample was run through Mini-PROTEAN TGX Precast Gels (Bio-Rad) and transferred onto Trans-Blot Turbo Mini 0.2 μm nitrocellulose membranes (Bio-Rad, 1704158) using Trans-Blot Turbo Transfer apparatus (Bio-Rad). Membrane was stained with primary antibodies anti-α-Tubulin (dilution 1:1000), anti-p44/42 MAPK (dilution 1:1000) and anti-phospho-p44/42 MAPK (dilution 1:1000) diluted in Odyssey blocking buffer overnight at 4°C. Secondary antibodies donkey anti-rabbit (dilution 1:5000) and donkey anti-mouse (dilution 1:5000) were applied for 1 h at RT. Blots were scanned with an Odyssey imaging system (LI-COR Biosciences) and quantified using the ImageJ software.

### Quantification and statistical analysis

All statistical evaluations (unpaired, two-tailed Student’s *t*-tests or ANOVA test) were done using GraphPad Prism 5.0. Experiments were done in the number of replicates as indicated for each figure.

## Supplemental information

Document S1. Figures S1–S7 and Table S2

Table S1. Detailed clinical history of patients from cohort 1, cohort 2, cohort 3, cohort 4, and cohort 5, related to Figures 1–3 and 5–6

Document S2. Article plus supplemental information
