# Supplementary material for: Monitoring melanoma patients on treatment reveals a distinct macrophage population driving targeted therapy resistance
Source: Cell Rep Med. 2024 Jun 27;5(7):101611. doi: 10.1016/j.xcrm.2024.101611 (PMC11293307; doi:10.1016/j.xcrm.2024.101611)
Supplement: Document S1. Figures S1–S7 and Table S2 [file mmc1.pdf]

Cell Reports Medicine, Volume *5*

Supplemental information

Monitoring melanoma patients on treatment reveals a distinct macrophage population driving targeted therapy resistance

Jelena Vasilevska, Phil Fang Cheng, Julia Lehmann, Egle Ramelyte, Julia Martínez Gómez, Florentia Dimitriou, Federica Sella, TuPro Consortium, Daria Ferretti, Adrian Salas-Bastos, Whitney Shannon Jordaan, Mitchell Paul Levesque, Reinhard Dummer, and Lukas Sommer

Supplementary Materials for

Monitoring melanoma patients on treatment reveals a distinct macrophage population driving targeted therapy resistance

Jelena Vasilevska et al.

Corresponding author: Lukas Sommer, lukas.sommer@anatomy.uzh.ch

The Supplementary Materials include:

Supplementary Figures S1 to S7

Supplementary Table S2

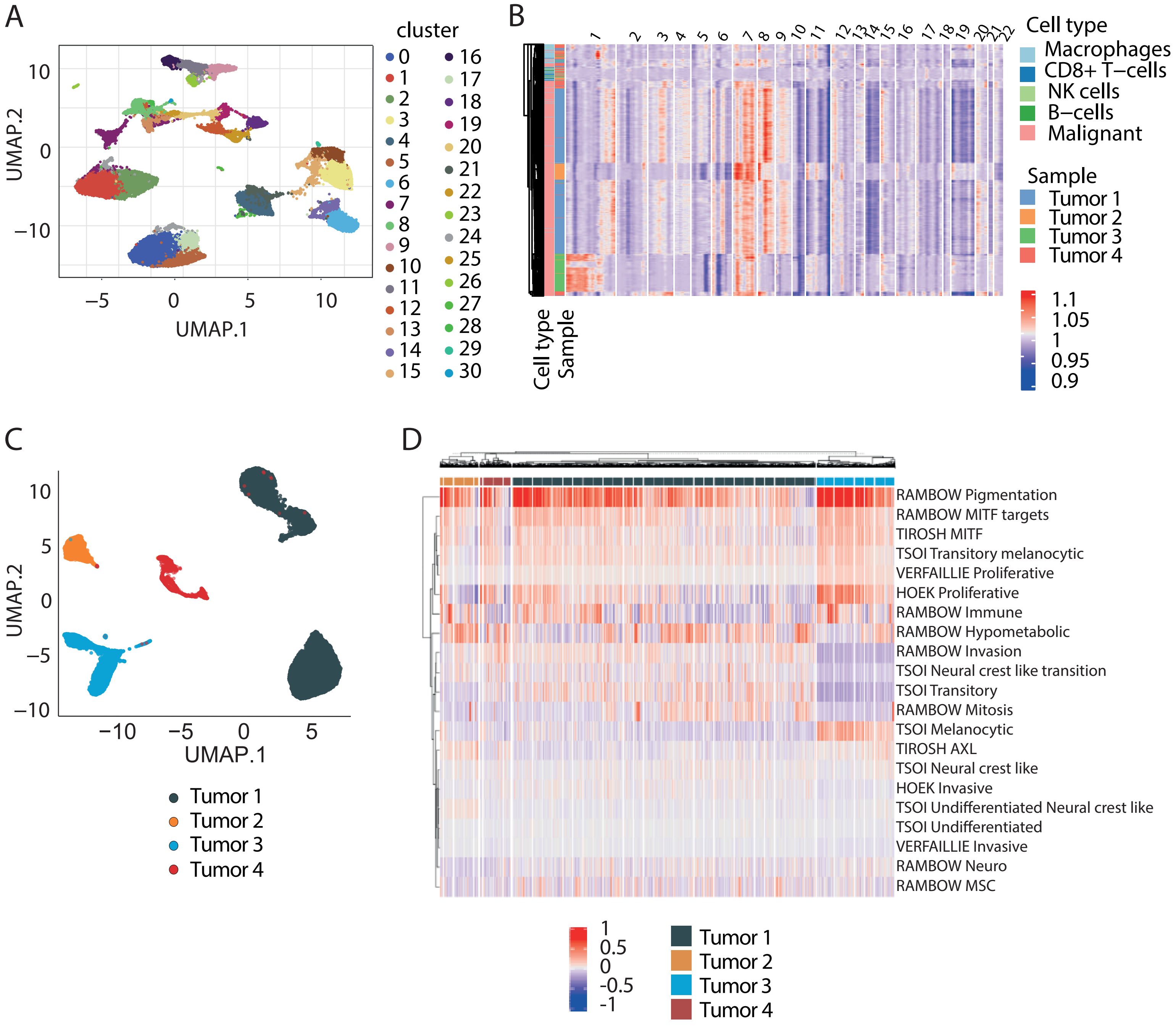
A
B
C
D
UMAP.2
UMAP.1
cluster
0
1
2
3
4
5
6
7
8
9
10
11
12
13
14
15
16
17
18
19
20
21
22
23
24
25
26
27
28
29
30
Cell type
Sample
Macrophages
CD8+ T−cells
NK cells
B−cells
Malignant
Tumor 1
Tumor 2
Tumor 3
Tumor 4
1.1
1.05
1
0.95
0.9
RAMBOW Pigmentation
RAMBOW MITF targets
TIROSH MITF
TSOI Transitory melanocytic
VERFAILLIE Proliferative
HOEK Proliferative
RAMBOW Immune
RAMBOW Hypometabolic
RAMBOW Invasion
TSOI Neural crest like transition
TSOI Transitory
RAMBOW Mitosis
TSOI Melanocytic
TIROSH AXL
TSOI Neural crest like
HOEK Invasive
TSOI Undifferentiated Neural crest like
TSOI Undifferentiated
VERFAILLIE Invasive
RAMBOW Neuro
RAMBOW MSC
1
0.5
0
-0.5
-1

Figure S1. **Cell populations detected by scRNAseq in Cohort 1 and its characterization. Related to Figure 1 and 2.** (A) UMAP plot showing 31 clusters in the tumors of Cohort 1 after PCA and unsupervised Louvain-based clustering. (B) Heatmap of copy number analysis of tumors from Cohort 1 by InferCNV. $CD4^+$ T-cells were used as reference. (C) UMAP plot represents clustering of malignant cells from tumors of Cohort 1. (D) Heatmap showing the enrichment scores of gene signatures of malignant cell populations from tumors of Cohort 1 with published resistance-related phenotypic markers.

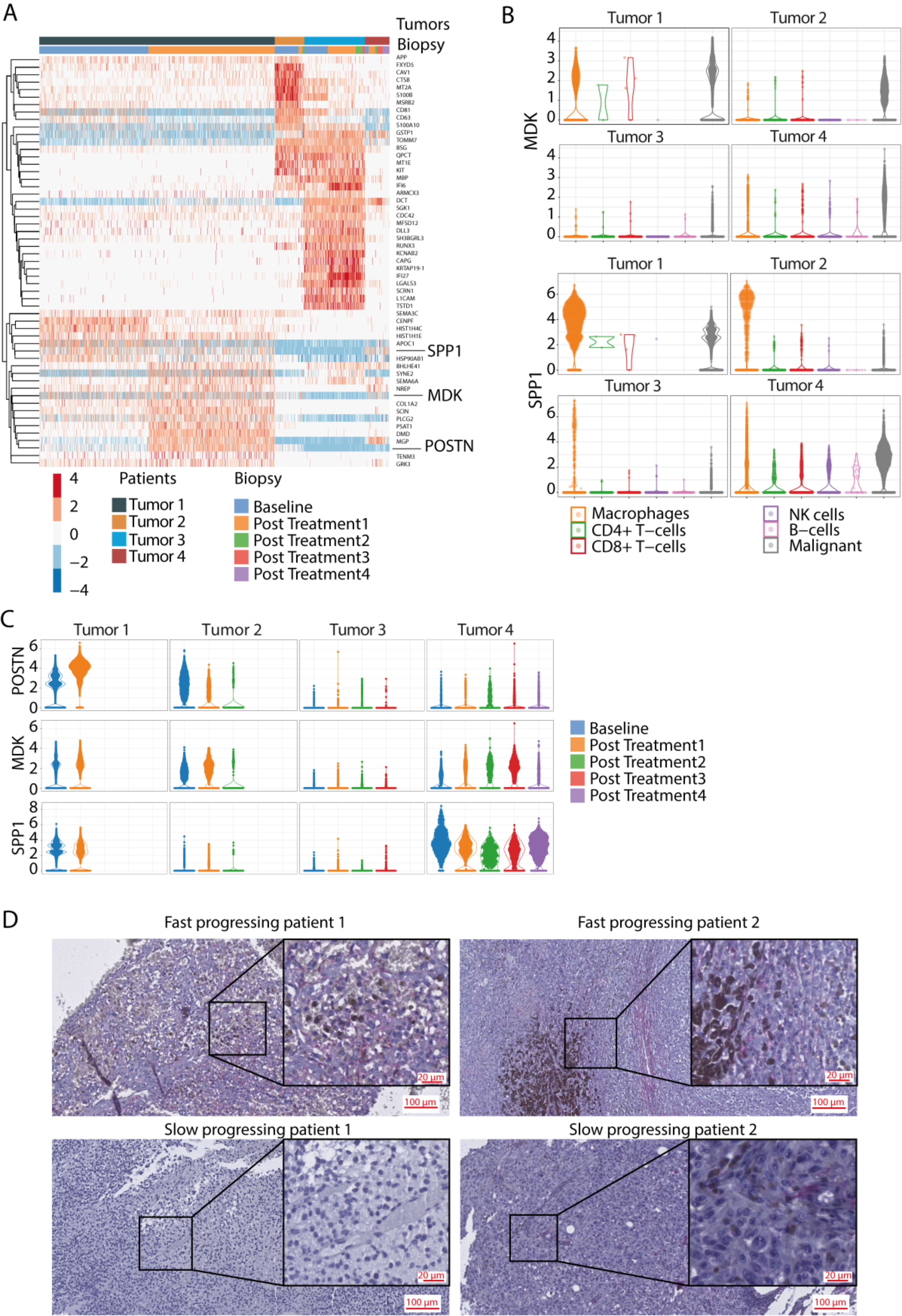

A
Tumors
Biopsy
APP
FXYD5
CAV1
CTSB
MT2A
S100B
MSRB2
CD81
CD63
S100A10
GSTP1
TOMM7
BSG
QPCT
MT1E
KIT
MBP
IFI6
ARMCX3
DCT
SGK1
CDC42
MFSD12
DLL3
SH3BGRL3
RUNX3
KCNAB2
CAPG
KRTAP19-1
IFI27
LGALS3
SCRN1
L1CAM
TSTD1
SEMA3C
CENPF
HIST1H4C
HIST1H1E
APOC1
SPP1
HSP90AB1
BHLHE41
SYNE2
SEMA6A
NREP
MDK
COL1A2
SCIN
PLCG2
PSAT1
DMD
MGP
POSTN
TENM3
GRK3
4
2
0
−2
−4
Patients
Tumor 1
Tumor 2
Tumor 3
Tumor 4
Biopsy
Baseline
Post Treatment1
Post Treatment2
Post Treatment3
Post Treatment4
B
Tumor 1
Tumor 2
Tumor 3
Tumor 4
MDK
SPP1
Macrophages
CD4+ T−cells
CD8+ T−cells
NK cells
B−cells
Malignant
C
Tumor 1
Tumor 2
Tumor 3
Tumor 4
POSTN
MDK
SPP1
Baseline
Post Treatment1
Post Treatment2
Post Treatment3
Post Treatment4
D
Fast progressing patient 1
Fast progressing patient 2
Slow progressing patient 1
Slow progressing patient 2
20 µm
100 µm

Figure S2. ***POSTN*, *MDK* and *SPP1* expression. Related to Figure 2.** (A) Heatmap indicating differential expressed genes in malignant cells of every tumor in Cohort 1 at every time point of biopsies. (B) Violin plots illustrating expression of *MDK* and *SPP1* in the cell populations for each tumor in Cohort 1 identified by scRNAseq. (C) Violin plots showing expression of *POSTN*, *MDK* and *SPP1* in malignant cells at every FNA time point for each tumor in Cohort 1. (D) Representative pictures of POSTN IHC staining in fast and slow progressing tumors.

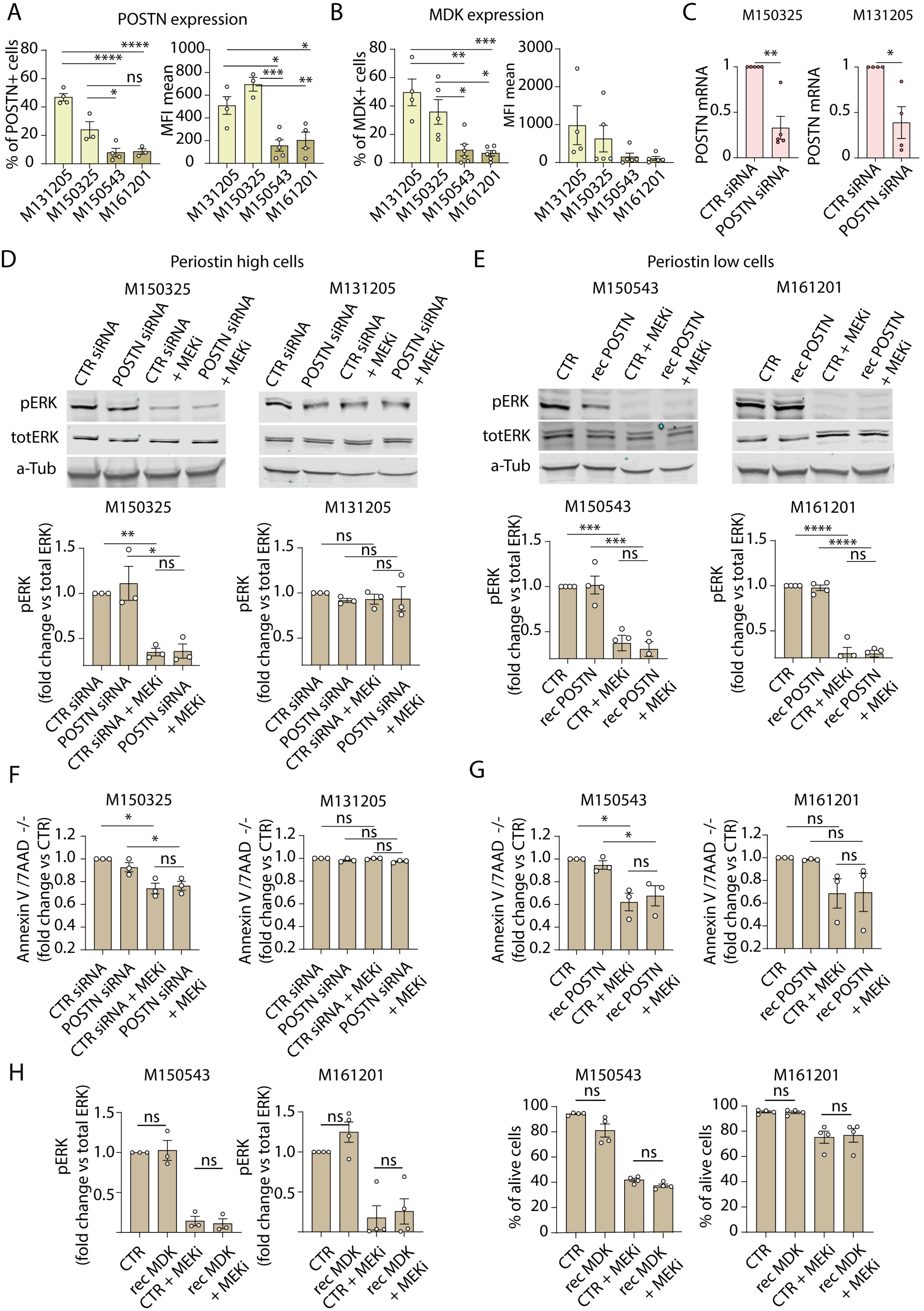
A
POSTN expression
% of POSTN+ cells
MFI mean
M131205
M150325
M150543
M161201
B
MDK expression
% of MDK+ cells
MFI mean
C
M150325
M131205
POSTN mRNA
CTR siRNA
POSTN siRNA
D
Periostin high cells
M150325
M131205
CTR siRNA
POSTN siRNA
CTR siRNA + MEKi
POSTN siRNA + MEKi
pERK
totERK
a-Tub
pERK (fold change vs total ERK)
E
Periostin low cells
M150543
M161201
CTR
rec POSTN
CTR + MEKi
rec POSTN + MEKi
F
M150325
M131205
Annexin V /7AAD -/- (fold change vs CTR)
G
M150543
M161201
H
M150543
M161201
CTR
rec MDK
CTR + MEKi
rec MDK + MEKi
% of alive cells
ns

Figure S3. **POSTN and MDK could not regulate resistance formation *in vitro*. Related to Figure 2.** (A) POSTN expression in melanoma cells (n=3-4 independent experiments, ordinary one-way ANOVA test, mean ± s.e.m). (B) MDK expression in melanoma cells (n=4-6 independent experiments, ordinary one-way ANOVA test, mean ± s.e.m). (C) Down-regulation of *POSTN* expression in melanoma cells after cell transfection with POSTN siRNA. The analysis was done with qRT-PCR. The data are normalized to expression of the housekeeping gene *PPIA* and presented as fold change to CTR siRNA (n=4-5 independent experiments, unpaired t-test, mean ± s.e.m). (D-E) Representative western blots of MAPK signaling pathway (pERK and total (tot)ERK antibody) after (D) POSTN expression was inhibited in cells via POSTN siRNA transfection for 24h and then treated with MEKi for 3 days and (E) cells were pretreated with recombinant (rec) POSTN for 24h and then treated with MEKi for 3 days. α-Tubulin antibody was used as loading control. Graphical analysis shows a quantification of pERK expression. Quantification was performed by ImageJ software. Results were normalized to totERK and quantified as fold change versus loading control α-Tubulin (n=3-4 independent experiments, ordinary one-way ANOVA test, mean ± s.e.m). (F-G) Bar graphs showing percentage of alive cells analyzed by flow cytometry data (Annexin V-negative, 7AAD-negative) after (F) POSTN expression was inhibited in cells via POSTN siRNA transfection for 24h and then treated with MEKi for 3 days and (G) Cells were pretreated with recombinant (rec) POSTN for 24h and then treated with MEKi for 3 days (n=3 independent experiments, ordinary one-way ANOVA test, mean ± s.e.m). (H) Bar graphs showing quantification of pERK expression and percentage of alive cells analyzed by flow cytometry data (Annexin V-negative, 7AAD-negative) after cells were pretreated with recombinant (rec) MDK for 24h and then treated with MEKi for 3 days. For the western blot, α-Tubulin antibody was used as loading control. Quantification was performed by ImageJ software. Results were normalized to total (tot)ERK and quantified as fold change versus loading control α-Tubulin. (n=3-4 independent experiments, ordinary one-way ANOVA test, mean ± s.e.m).

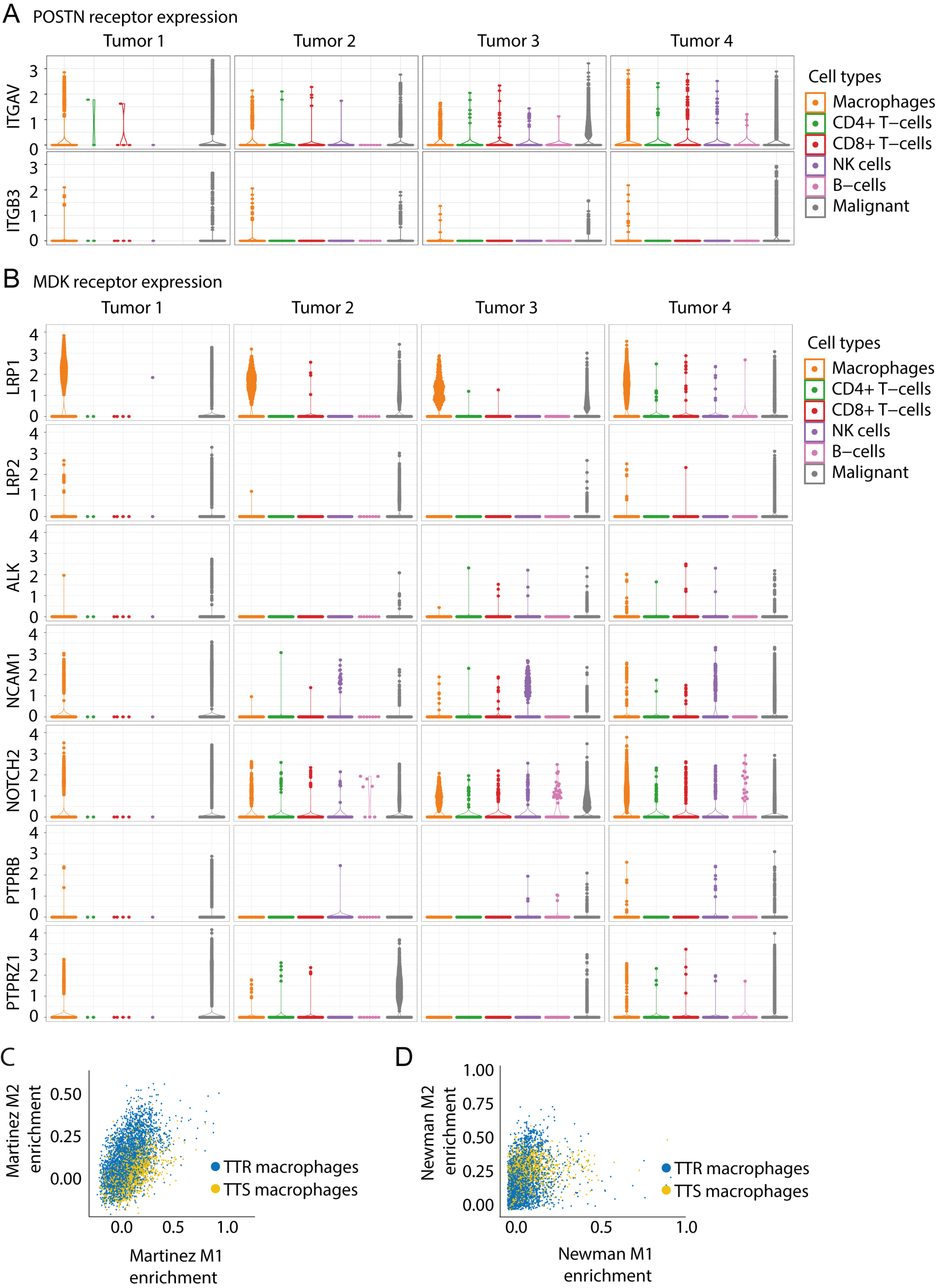
A POSTN receptor expression
Tumor 1
Tumor 2
Tumor 3
Tumor 4
ITGAV
ITGB3
Cell types
Macrophages
CD4+ T−cells
CD8+ T−cells
NK cells
B−cells
Malignant
B MDK receptor expression
LRP1
LRP2
ALK
NCAM1
NOTCH2
PTPRB
PTPRZ1
C
Martinez M2 enrichment
Martinez M1 enrichment
TTR macrophages
TTS macrophages
D
Newman M2 enrichment
Newman M1 enrichment

Figure S4. **Expression of POSTN and MDK receptors and analysis of TTR and TTS macrophage cell types with classical M1 and M2 markers. Related to Figure 3.** (A) CellChat analysis showing expression of POSTN receptors across tumors and cell populations of Cohort 1. (B) CellChat analysis showing expression of MDK receptors across tumors and cell populations of Cohort. (C) TTR and TTS macrophage module score for Martinez M1 and M2 macrophage gene signatures. (D) TTR and TTS macrophage module score for Newman M1 and M2 macrophage gene signatures.

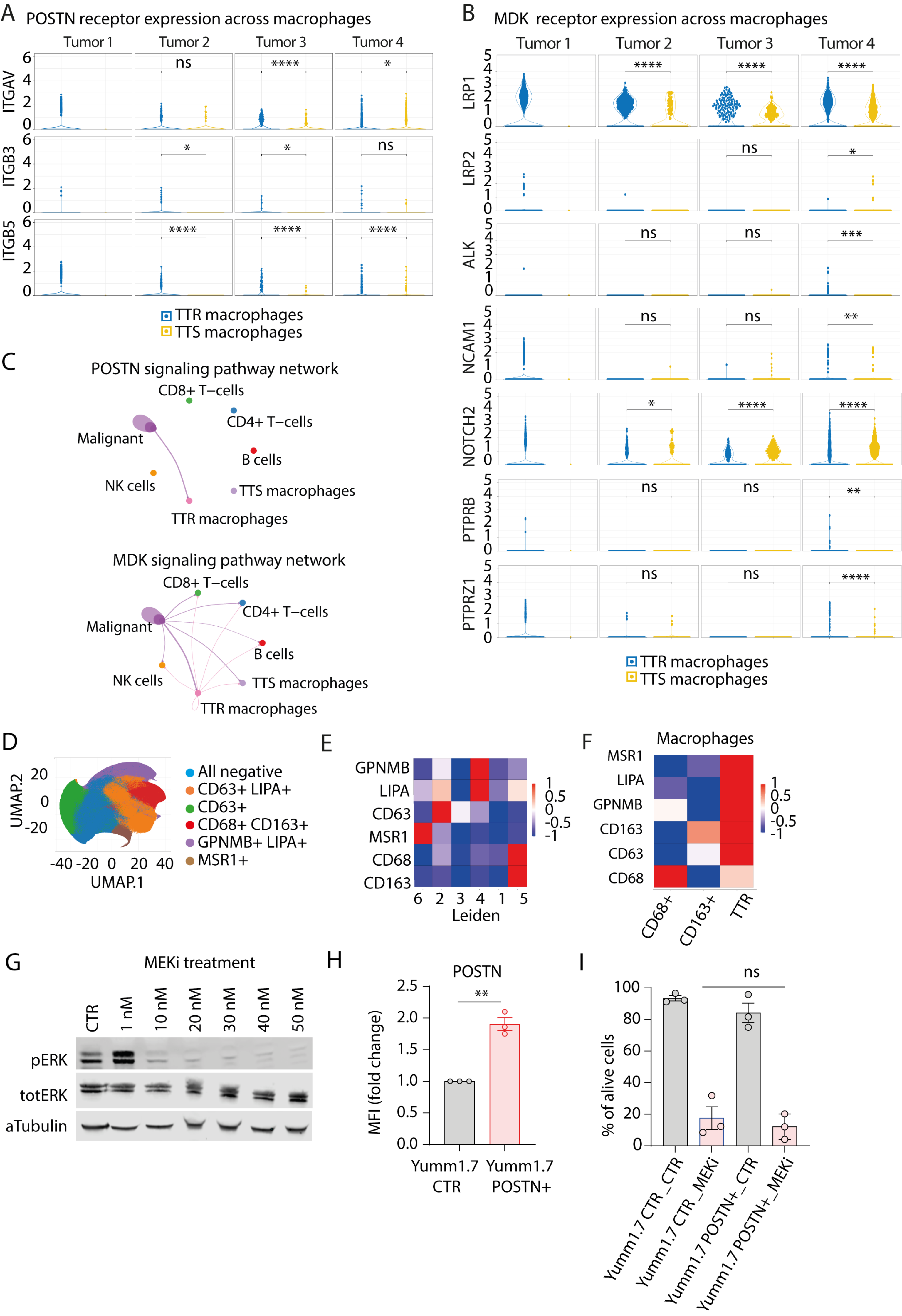
A
POSTN receptor expression across macrophages
Tumor 1
Tumor 2
Tumor 3
Tumor 4
ITGAV
ITGB3
ITGB5
ns
****
*
TTR macrophages
TTS macrophages
B
MDK receptor expression across macrophages
LRP1
LRP2
ALK
NCAM1
NOTCH2
PTPRB
PTPRZ1
***
**
C
POSTN signaling pathway network
CD8+ T−cells
CD4+ T−cells
Malignant
B cells
NK cells
TTS macrophages
TTR macrophages
MDK signaling pathway network
D
UMAP.2
UMAP.1
All negative
CD63+ LIPA+
CD63+
CD68+ CD163+
GPNMB+ LIPA+
MSR1+
E
GPNMB
LIPA
CD63
MSR1
CD68
CD163
Leiden
F
Macrophages
CD68+
CD163+
TTR
G
MEKi treatment
CTR
1 nM
10 nM
20 nM
30 nM
40 nM
50 nM
pERK
totERK
aTubulin
H
POSTN
MFI (fold change)
Yumm1.7 CTR
Yumm1.7 POSTN+
I
% of alive cells
Yumm1.7 CTR _CTR
Yumm1.7 CTR _MEKi
Yumm1.7 POSTN+_CTR
Yumm1.7 POSTN+_MEKi

Figure S5. **POSTN and MDK receptor expression across TTR and TTS macrophages, their signaling pathway networks, and characterization of TTR macrophages. Related to Figure 3 and 4.** (A) CellChat analysis showing expression of POSTN receptors between TTR (blue) and TTS macrophages (yellow) (Wilcoxon rank sum test) of Cohort 1. (B) CellChat analysis showing expression of MDK receptors between TTR (blue) and TTS macrophages (yellow) (Wilcoxon rank sum test) of Cohort 1. (C) POSTN and MDK signaling pathway network in Cohort 1. Malignant cells were used as senders and other cell populations used as receivers. (D) UMAP plot showing 6 clusters after multiplex immunohistochemistry staining of TTR and other macrophage markers in Cohort 3 generated by Leiden clustering. (E) Heatmap showing the average marker expression per Leiden cluster in Cohort 3. (F) Heatmap showing the average marker expression per subcluster of Leiden cluster 5 in Cohort 3. (G) Representative western blots of MAPK signaling pathway (pERK and total (tot)ERK antibody) in Yumm1.7 cell line after cells were treated with different concentrations of MEKi for 3 days. α-Tubulin antibody was used as loading control.(H) Expression of POSTN in Yumm1.7 cells infected with empty lentiviral vector (Yumm1.7 CTR) or POSTN overexpressing lentiviral vector (Yumm1.7 POSTN+) (flow cytometry data, n=3 independent experiments, unpaired t test, mean ± s.e.m). (I) Bar graphs show cell survival of Yumm1.7 CTR and Yumm1.7 POSTN+ cells after treatment with MEKi for 3 days (flow cytometry, Annexin V-negative, 7AAD-negative) (n=3 independent experiments, ordinary one-way ANOVA test, mean ± s.e.m).

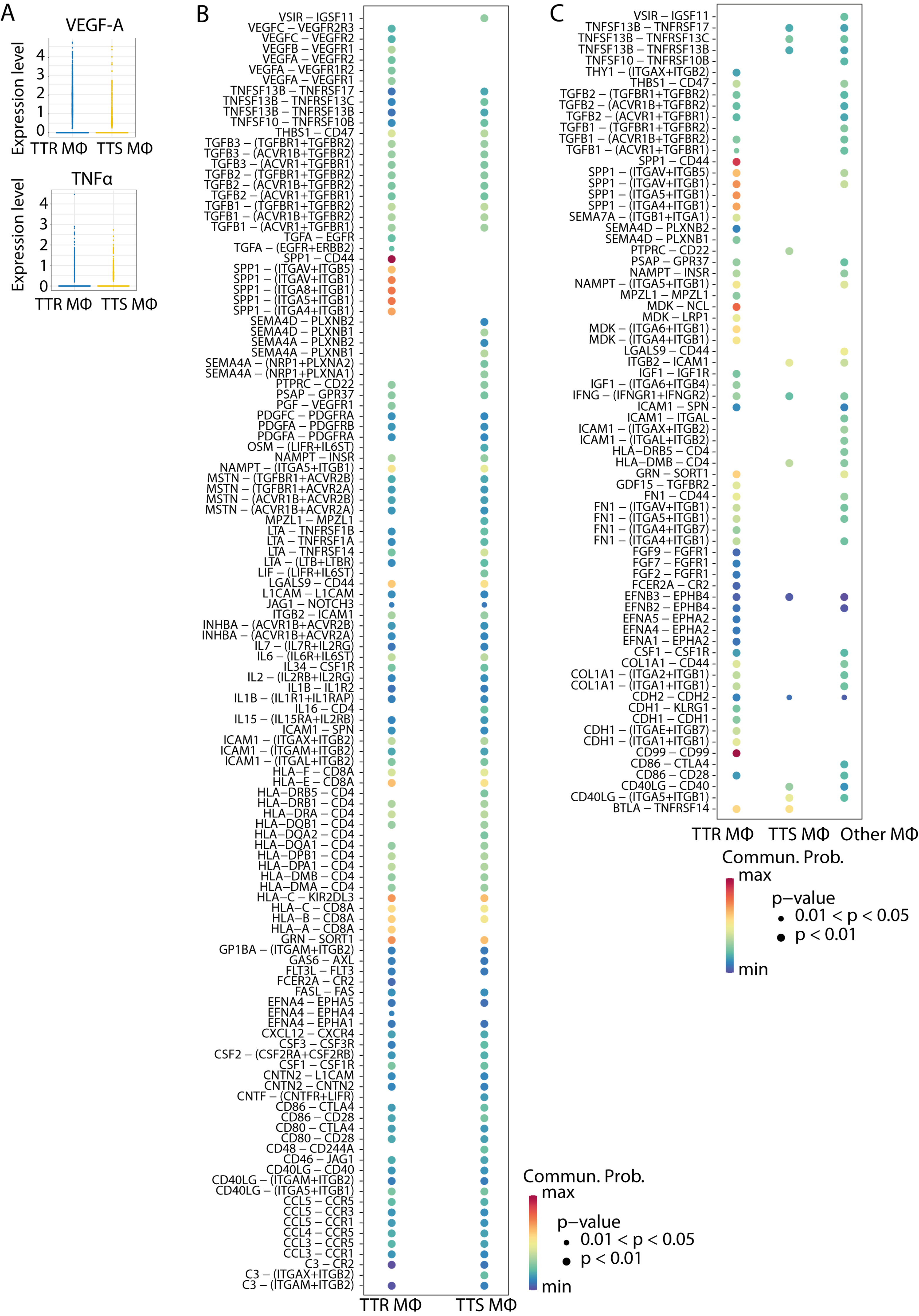
A
VEGF-A
TNFα
Expression level
TTR MΦ
TTS MΦ
B
C
Other MΦ
Commun. Prob.
max
min
p−value
0.01 < p < 0.05
p < 0.01

Figure S6. **Ligand-receptor-based CellChat communication of TTR and TTS macrophages with malignant cells. Related to Figure 7.** (A) Expression of *VEGF-A* and *TNFα* in TTR and TTS macrophages. MΦ = macrophages.(B) Bubble plot showing ligand-receptor-based CellChat analysis of the FNA scRNAseq dataset from Cohort 1, with TTR and TTS macrophages as senders and the malignant cells as receivers. MΦ = macrophages. (C) Bubble plot showing ligand-receptor based CellChat analysis of Cohort 5 with the TTR, TTS and other macrophages as senders and the malignant cells as receivers. MΦ = macrophages.

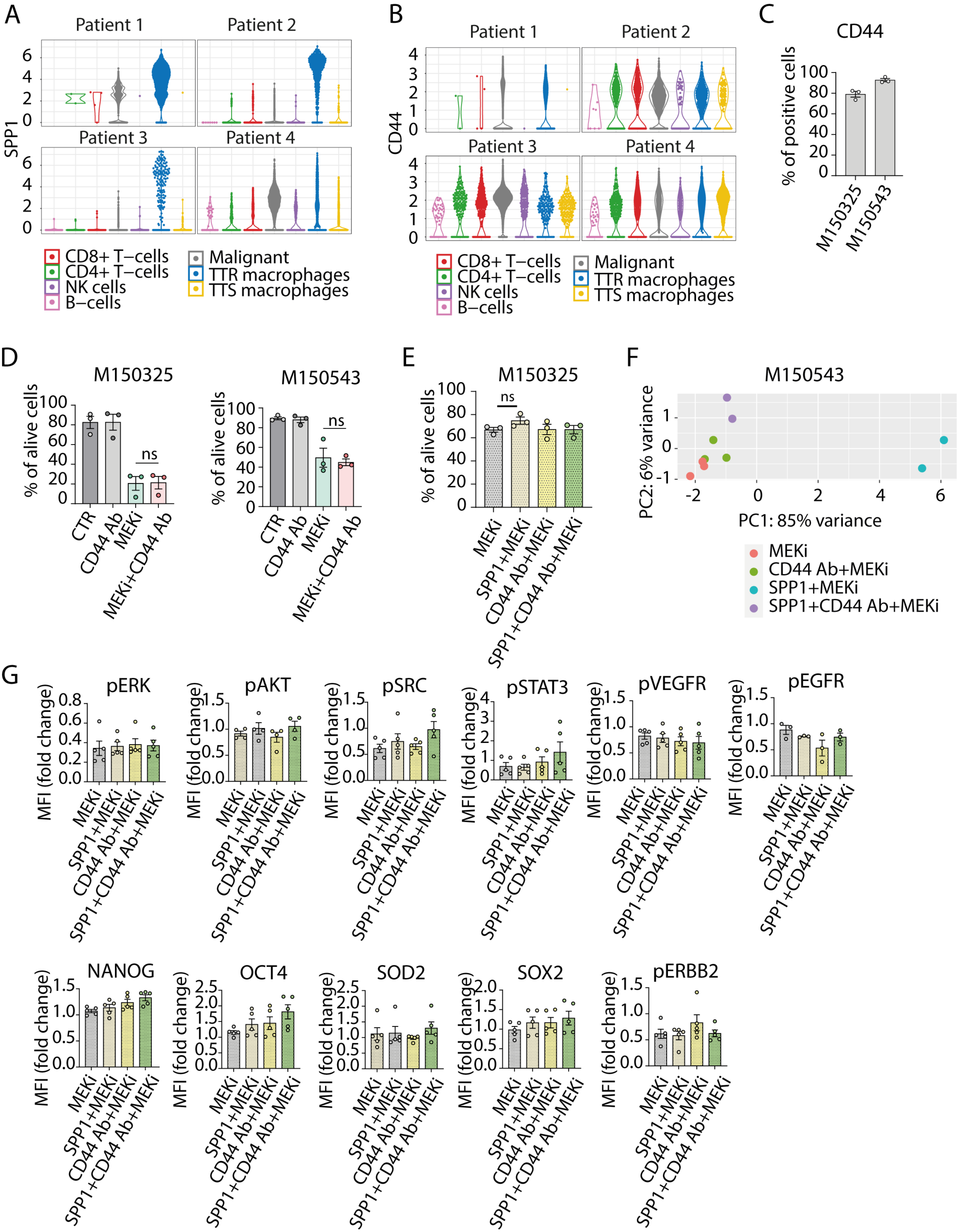

A
Patient 1
Patient 2
Patient 3
Patient 4
SPP1
CD8+ T−cells
CD4+ T−cells
NK cells
B−cells
Malignant
TTR macrophages
TTS macrophages
B
Patient 1
Patient 2
Patient 3
Patient 4
CD44
CD8+ T−cells
CD4+ T−cells
NK cells
B−cells
Malignant
TTR macrophages
TTS macrophages
C
CD44
% of positive cells
M150325
M150543
D
M150325
M150543
% of alive cells
ns
CTR
CD44 Ab
MEKi
MEKi+CD44 Ab
E
M150325
% of alive cells
ns
MEKi
SPP1+MEKi
CD44 Ab+MEKi
SPP1+CD44 Ab+MEKi
F
M150543
PC2: 6% variance
PC1: 85% variance
MEKi
CD44 Ab+MEKi
SPP1+MEKi
SPP1+CD44 Ab+MEKi
G
pERK
pAKT
pSRC
pSTAT3
pVEGFR
pEGFR
NANOG
OCT4
SOD2
SOX2
pERBB2
MFI (fold change)
MEKi
SPP1+MEKi
CD44 Ab+MEKi
SPP1+CD44 Ab+MEKi

Figure S7. ***SPP1* and *CD44* expression and their related pathways. Related to Figure 7**. (A) Violin plots showing expression of *SPP1* across the tumors and cell populations identified by scRNAseq of Cohort 1. (B) Violin plots showing expression of *CD44* across the tumors and different cell populations of Cohort 1. (C) CD44 expression in melanoma cells (flow cytometry data, n=3 independent experiments, mean ± s.e.m). (D) Bar graphs show cell survival after treatment with MEKi or treatment with MEKi following a 2 hour pretreatment with CD44 blocking antibody (flow cytometry, Annexin V-negative, 7AAD-negative) (n=3 independent experiments, ordinary one-way ANOVA test, mean ± s.e.m). (E) Bar graphs show cell survival after treatment with MEKi for 3 days following a 2-hour pretreatment with recombinant SPP1 or CD44 blocking antibody or combined recombinant SPP1+ CD44 blocking antibody (flow cytometry, Annexin V-negative, 7AAD-negative) (n=3 independent experiments, ordinary one-way ANOVA test, mean ± s.e.m). (F) Principal component analysis (PCA) plot for the transcriptomic data derived from the four experimental groups. M150543 cells were treated with MEKi for 20 hours following a 2 hour pretreatment with recombinant SPP1 or CD44 blocking antibody or combined recombinant SPP1+ CD44 blocking antibody. Points are color coded per treatment (MEKi = 3 N; CD44Ab + MEKi = 3 N; SPP1 + MEKi = 2 N; SPP1 + CD44Ab + MEKi = 2 N). (G) Quantification of canonical CD44 downstream signaling pathways. M150543 cells were treated with MEKi alone for 6 hours or following a 2-hour pretreatment with CD44 blocking antibody, recombinant SPP1 or combined recombinant SPP1+ CD44 blocking antibody (flow cytometry, n=4-5 independent experiments, ordinary one-way ANOVA test, mean ± s.e.m).

Table S2

REAGENT or RESOURCE	SOURCE	IDENTIFIER
**qRT-PCR primers**		
Human *POSTN* forward primer: GCTATTCTGACGCCTCAAAACT	Microsynth	N/A
Human *POSTN* reverse primer: AGCCTCATTACTCGGTGCAAA	Microsynth	N/A
Human *PPIA* forward primer: TTCATCTGCACTGCCAAGAC	Microsynth	N/A
Human *PPIA* reverse primer: TCGAGTTGTCCACAGTCAGC	Microsynth	N/A
Human *SPP1* forward primer: CTCCATTGACTCGAACGACTC	Microsynth	N/A
Human *SPP1* reverse primer: CAGGTCTGCGAAACTTCTTAGAT	Microsynth	N/A
Mouse *SPP1* forward primer: AGCAAGAAACTCTTCCAAGCAA	Microsynth	N/A
Mouse *SPP1* reverse primer: GTGAGATTCGTCAGATTCATCCG	Microsynth	N/A
Mouse *GAPDH* forward primer: ACTCCACTCACGGCAAATTC	Microsynth	N/A
Mouse *GAPDH* reverse primer: TCTCCATGGTGGTGAAGACA	Microsynth	N/A

Table S2. **qRT-PCR primers. Related to STAR Methods.**
